# Protein O‐GlcNAcylation in Health and Diseases

**DOI:** 10.1002/mco2.70536

**Published:** 2025-12-10

**Authors:** Zhihong Ran, Chuanbao Chen, Jingfeng Ou, Guanyi Wu, Chao Yang, Xiaoyou Liu

**Affiliations:** ^1^ Department of Organ Transplantation The First Affiliated Hospital of Guangzhou Medical University Guangzhou China

**Keywords:** biological processes, diseases, health, mechanisms, O‐GlcNAcylation, therapy

## Abstract

O‐GlcNAcylation is a reversible posttranslational modification of proteins that has garnered significant attention in recent years. By regulating the structure and function of proteins, it plays a critical role in various biological processes. Normal O‐GlcNAcylation is essential for maintaining internal homeostasis and is involved in controlling fundamental biological events such as gene expression, the cell cycle regulation, metabolism, and signal transduction. Conversely, aberrant O‐GlcNAcylation is closely linked to the onset and progression of various diseases—including neurodegenerative diseases, cancers, cardiovascular diseases, and immune‐related diseases—where it drives pathological development. Currently, there is a lack of comprehensive reviews systematically addressing the specific mechanisms of O‐GlcNAcylation under both physiological and pathological conditions. Therefore, this article aims to summarize its dual role in maintaining organismal homeostasis and promoting disease pathogenesis, providing an integrated evaluation of the biological significance of this modification in health and diseases. Furthermore, it discusses the potential of O‐GlcNAcylation as a therapeutic target, explores its clinical applications, and analyzes the current challenges and future directions in drug development, thereby offering theoretical insights and research perspectives for related fields.

## Graphical abstract

We first introduced the beneficial effects of normal protein O‐GlcNAcylation on health, impacting biological processes such as gene expression, cell cycle progression, metabolism, and signal transduction. Next, we described the role of abnormal protein O‐GlcNAcylation in the development and progression of diseases, including neurodegenerative diseases, cancers, cardiovascular diseases, and immune‐related diseases. Finally, we explored the feasibility of targeting O‐GlcNAcylation for therapeutic purposes and discussed current challenges and future directions, aiming to provide new insights for drug development and the optimization of clinical treatment strategies.

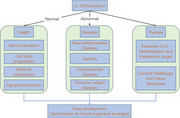

## 1 Introduction

**FIGURE 1:**
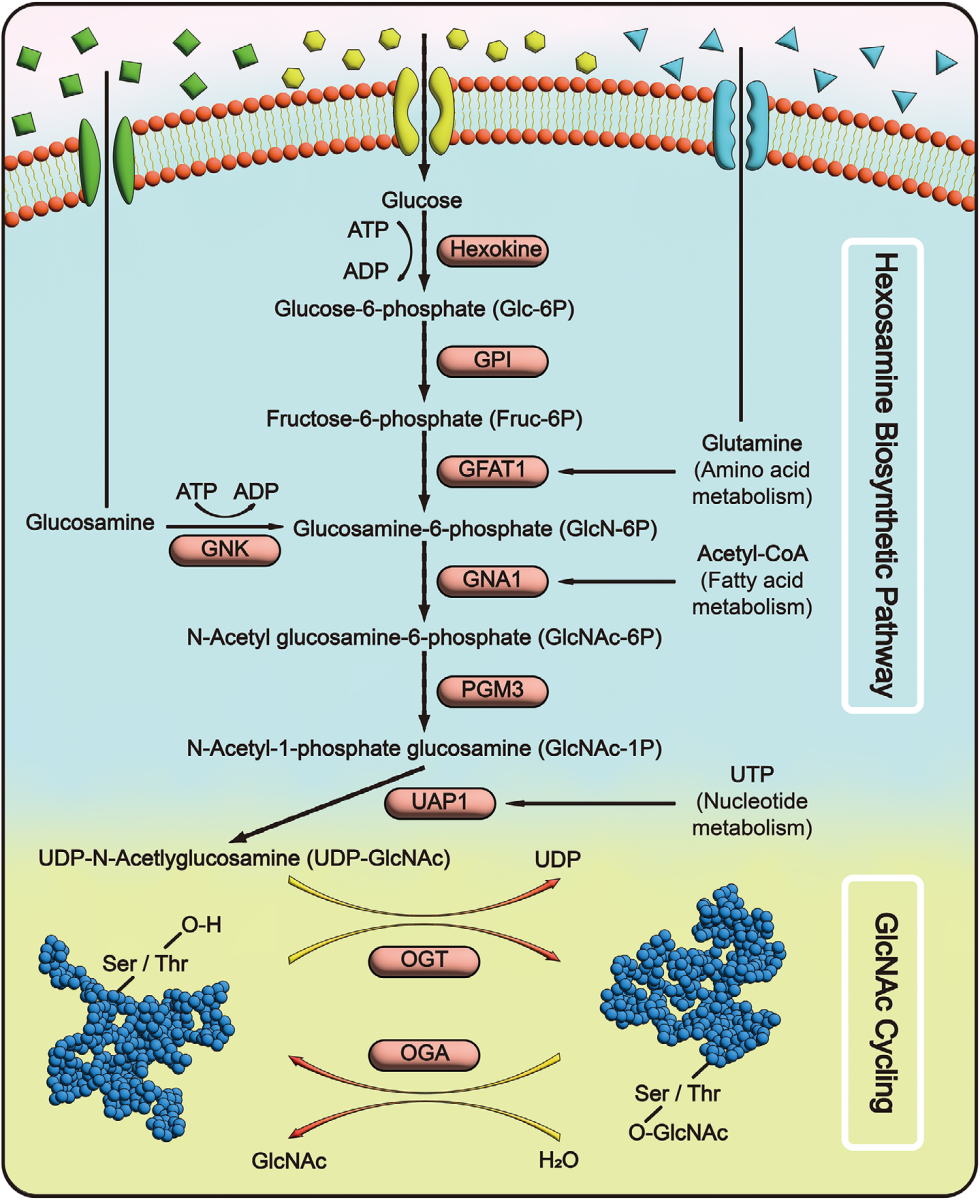
Flow diagram of HBP and GlcNAc cycling. Glucose entering the cell is catalyzed by hexokinase to form glucosamine‐6‐phosphate (GlcN‐6P), which is later isomerized to fructose‐6‐phosphate (Fruc‐6P) by the enzyme GPI. GFAT1 is the rate‐limiting enzyme of HBP. Fruc‐6P binds catalytically to the biomolecules glutamine, glucosamine, and acetyl‐CoA via GFAT1 and GNA1 to sequentially produce glucosamine‐6‐phosphate (GlcN‐6P) and N‐acetyl glucosamine‐6‐phosphate (GlcNAc‐6P). Posterior GlcNAc‐6P isomerization to N‐acetylglucosamine 1‐phosphate (GlcNAc‐1P) catalyzed by PGM3. With the participation of UTP, GlcNAc‐1P is catalyzed by UAP1 to produce the end product of HBP, uridine diphosphate‐N‐acetylglucosamine (UDP‐GlcNAc). Finally, UDP‐GlcNAc was used as a substrate to complete the O‐GlcNAcylation and de‐O‐GlcNAcylation modification of the target proteins by the combined action of the enzymes OGT and OGA. *Abbreviations*: GPI: glucose‐6‐phosphate isomerase; GFAT1: glutamine‐fructose‐6‐phosphate aminotransferase 1; GNK: GlcNAc kinase; GNA1: d‐glucosamine‐6‐phosphate N‐acetyltransferase 1; PGM3: phosphoglucomutase 3; UAP1: UDP‐N‐acetylhexosamine pyrophosphorylase 1; UTP: uridine triphosphate; UDP: uridine diphosphate; OGT: O‐GlcNActransferase; OGA: O‐GlcNAcase.

O‐GlcNAcylation was first discovered in 1980 during research on cell surface glycan structures [1]. As a dynamic and reversible posttranslational modification, it occurs primarily on serine/threonine (Ser/Thr) residues of proteins and is regulated by only two counteracting enzymes: O‐GlcNAc transferase (OGT) and O‐GlcNAc hydrolase (OGA) [2, 3, 4, 5]. As a glycosyltransferase, OGT catalyzes the covalent attachment of the “GlcNAc” moiety from UDP‐GlcNAc to Ser/Thr residues of target proteins, thereby mediating O‐GlcNAcylation. In contrast, OGA hydrolyzes this glycosidic bond to remove the “GlcNAc” modification, facilitating de‐O‐GlcNAcylation [6, 7]. Both OGT and OGA are evolutionarily highly conserved [8, 9, 10, 11], and their genetic deletion leads to developmental defects or even lethality [12, 13], indicating their indispensable roles in maintaining the dynamic balance of protein O‐GlcNAcylation and ensuring structural and functional stability of proteins. The substrate for O‐GlcNAcylation, UDP‐GlcNAc, is mainly produced through the hexosamine biosynthesis pathway (HBP) [14] (Figure 1). Approximately 2–5% of glucose in the body enters this pathway, where it is metabolized along with glutamine, acetyl coenzyme A (acetyl‐CoA) and uridine 5′‐triphosphate (UTP) to generate UDP‐GlcNAc [15]. Consequently, UDP‐GlcNAc is regarded as an intracellular “nutrient sensor” with its levels reflecting the nutritional and metabolic status of the organism [16]. To date, over 5000 proteins, including membrane, nuclear, cytoplasmic, and mitochondrial proteins, have been identified as O‐GlcNAcylation substrates [17, 18, 19], highlighting the broad and crucial role of this modification in regulating protein properties and functions. However, aberrant O‐GlcNAcylation can significantly alter the physicochemical properties and biological functions of proteins, thereby contributing to the development and progression of various diseases [20]. These findings demonstrate that O‐GlcNAcylation plays a critical role in both health and diseases.

Research has shown that knockout of the OGT gene has lethal effects on mouse embryos [21, 22] and accelerates cellular senescence and death [23, 24, 25], indicating that normal O‐GlcNAcylation is essential for embryonic development and cellular homeostasis. The health of an organism relies on the orderly execution of numerous biological processes, and O‐GlcNAcylation is extensively involved in these processes: it promotes mRNA transcription and protein translation; regulates transitions between the cell cycle phases (G1, S, G2, and M); influences the metabolism of substances; and participates in the activation and maintenance of various signaling molecules [26, 27]. Thus, O‐GlcNAcylation plays a central role in key biological processes such as gene expression, the cell cycle, metabolism, and signal transduction, serving as a fundamental mechanism for maintaining overall health.

Abnormal O‐GlcNAcylation has been confirmed as a critical factor in the pathogenesis of various diseases. In neurodegenerative diseases such as Alzheimer's disease (AD), Parkinson's disease (PD), Huntington's disease (HD), and amyotrophic lateral sclerosis (ALS), dysregulated expression of OGT and OGA in the brain leads to abnormal O‐GlcNAcylation levels, resulting in functional impairment of neuronal proteins and cellular damage [28]. In various malignant tumors, including lung, liver, colorectal, and breast cancers, elevated O‐GlcNAcylation levels significantly promote tumor cell proliferation, invasion, and metastasis [29]. Furthermore, abnormal O‐GlcNAcylation—either increased or decreased—is commonly observed in cardiovascular diseases (CVDs) [30] and immune‐related diseases [31, 32], accelerating disease progression. These findings indicate that dysregulated O‐GlcNAcylation markedly facilitates disease development.

It is evident that the state of normal and abnormal protein O‐GlcNAcylation is closely linked to health and disease. Although research on O‐GlcNAcylation has grown rapidly, comprehensive summaries remain relatively scarce. Therefore, this article first systematically reviews the important role of O‐GlcNAcylation in maintaining health, with an in‐depth discussion of its regulatory mechanisms in gene expression, the cell cycle, metabolism, and signal transduction. Second, it analyzes how aberrant O‐GlcNAcylation promotes the pathogenesis of neurodegenerative diseases, CVDs, cancers, and immune‐related diseases. Finally, the feasibility of targeting O‐GlcNAcylation as a clinical therapeutic strategy is evaluated, current challenges are discussed, and future research directions are proposed, providing a theoretical basis and constructive insights for the clinical management of related diseases.

## 2 O‐GlcNAcylation is an Important Maintainer of Health

O‐GlcNAcylation serves as an indispensable “vitamin” in the cellular life cycle, playing an essential role in orchestrating various biological processes and contributing significantly to the maintenance of internal homeostasis [33, 34]. This section will focus on how O‐GlcNAcylation promotes key biological events—such as gene expression, the cell cycle, metabolism, and signal transduction—and will elaborate in detail on its substantial contributions to overall organismal health.

### 2.1 O‐GlcNAcylation Promotes Gene Expression

O‐GlcNAcylation plays a crucial facilitatory role in the process of gene transcription [35]. Transcription—the synthesis of mRNA using a target gene as a template—is the key initial step in gene expression. The functions of numerous protein components involved in this process are precisely regulated by O‐GlcNAcylation. RNA polymerase II (RNAP II) is the central enzyme in transcription. O‐GlcNAcylation modifies Ser/Thr residues within its C‐terminal domain, which promotes the assembly of the preinitiation complex (PIC) and facilitates transcriptional elongation and termination [36, 37]. The formation of PIC is a critical event in transcription initiation. Studies have shown that inhibiting the enzymatic activity of OGT or OGA impedes PIC assembly, indicating that aberrant O‐GlcNAcylation levels significantly disrupt this process [38, 39]. Beyond RNAP II, proteomic analyses have identified 32 additional transcription‐related factors regulated by O‐GlcNAcylation. Dysregulation of this modification can directly lead to functional impairment of these factors, resulting in transcriptional arrest [37]. Histones, as the core components of chromatin structure, promote chromosome decondensation to form linear DNA templates when gene expression is required. O‐GlcNAcylation targets multiple sites on histone tails and acts as a histone modification itself [40, 41, 42, 43, 44, 45, 46, 47, 48, 49], thereby regulating processes like ubiquitination and methylation. This, in turn, promotes chromatin relaxation and facilitates gene transcription [48, 49]. Furthermore, the functions of many transcription‐related components depend on O‐GlcNAcylation, such as TATA‐binding protein (TBP) [50], topoisomerase I (Topo I) [51], and nucleoporins (NUPs) of the nuclear pore complex (NPC) [52]. In addition to directly modulating transcriptional components to promote gene expression, O‐GlcNAcylation also exerts indirect effects by influencing transcription factors. For example, the activity of HOXA1—a transcription factor belonging to the HOX family—is regulated by OGT, and its dysfunction can lead to transcriptional arrest [53, 54, 55, 56, 57, 58, 59]. The activation and stability of other key transcription factors, including Sp1 [60], Hnf‐1 [61], c‐Myc [62, 63], and serum response factor (SRF) [64], also rely on O‐GlcNAcylation [65]. In summary, O‐GlcNAcylation effectively promotes gene transcription by facilitating the assembly of transcriptional machinery and enhancing the activity and stability of transcription factors.

O‐GlcNAcylation plays a key regulatory role in mRNA splicing. As a central step in gene expression regulation, splicing generates multiple mRNA isoforms to enhance transcript diversity and increase the abundance of target mRNA. Studies show that the function of numerous splicing factors depends on O‐GlcNAcylation, which significantly affects accurate gene expression. Treatment of HEK‐293T cells with the OGT inhibitor OSMI‐2 markedly reduces the mRNA abundance of both OGT and OGA. This phenomenon is consistently observed in HCT116 colon cancer cells and mouse embryonic fibroblasts, indicating a conserved role of O‐GlcNAcylation in splicing regulation [66]. Ser/arginine‐rich protein kinase 2 (SRPK2), a key kinase in pre‐mRNA splicing [67, 68], can be O‐GlcNAcylated at Ser490, Thr492, and Thr498, thereby promoting its nuclear translocation and kinase activation [69]. In MCF7 breast cancer cells, reduced O‐GlcNAcylation decreases the mRNA abundance of genes involved in de novo fatty acid synthesis. Further studies confirm that this process is mediated through SRPK2‐regulated pre‐mRNA splicing, demonstrating that O‐GlcNAcylation influences mRNA maturation [69]. A similar mechanism exists in plants. AtACINUS in Arabidopsis, a homolog of mammalian splicing regulators, has been shown to undergo O‐GlcNAcylation [70]. Experiments indicate that ten AtACINUS‐dependent alternative splicing events strongly rely on O‐GlcNAcylation levels, highlighting its important role in plant splicing regulation. Additionally, O‐GlcNAcylation affects transcription and splicing factor activity by modulating AtACINUS, further regulating gene expression [70]. Notably, O‐GlcNAcylation plays a critical role in splicing regulation associated with various diseases. For example, both TDP‐43 [71] (linked to neurodegenerative diseases) and AUF1 [72] (associated with ovarian cancer) are regulated by O‐GlcNAcylation, and aberrant levels lead to splicing dysregulation and disease pathogenesis. In summary, abnormal O‐GlcNAcylation disrupts the function and modification of splicing factors, resulting in pre‐mRNA splicing dysregulation. In contrast, appropriate and precise O‐GlcNAcylation promotes normal pre‐mRNA splicing, ensures accurate and stable gene expression, and plays an essential protective role in maintaining normal cellular physiological functions.

O‐GlcNAcylation plays a key regulatory role in protein translation—the process by which mRNA, transcribed from genes, serves as a template for protein synthesis with the assistance of ribosomes, tRNA, and associated factors. Multiple studies have confirmed that numerous translation factors and the ribosomes themselves are regulated by O‐GlcNAcylation [73]. To date, more than 20 core ribosomal proteins are known to be modulated by this modification, highlighting its close association with the protein translation machinery [74]. Furthermore, stress granules (SGs) and processing bodies (PBs), which are important components of ribonucleoprotein (RNP) granules, are involved in the regulation of mRNA translation and decay. Research indicates that aberrant O‐GlcNAcylation disrupts the proper assembly of SGs and PBs, thereby suppressing protein translation [75, 76, 77, 78, 79]. Additionally, ribosomal protein subunits such as RPL5, RPL10, RPL11, and RPL13a have been identified as targets of O‐GlcNAcylation. Dysregulation of this modification can significantly impair ribosomal function and hinder protein synthesis [80]. Although further investigation is needed to understand how O‐GlcNAcylation regulates other elements within the translation process, its profound impact on ribosomal function is well established. O‐GlcNAcylation is involved in multiple aspects of ribosomal activity, and as the central machinery for protein synthesis, the proper functioning of ribosomes is critically dependent on precise regulation by O‐GlcNAcylation.

**FIGURE 2:**
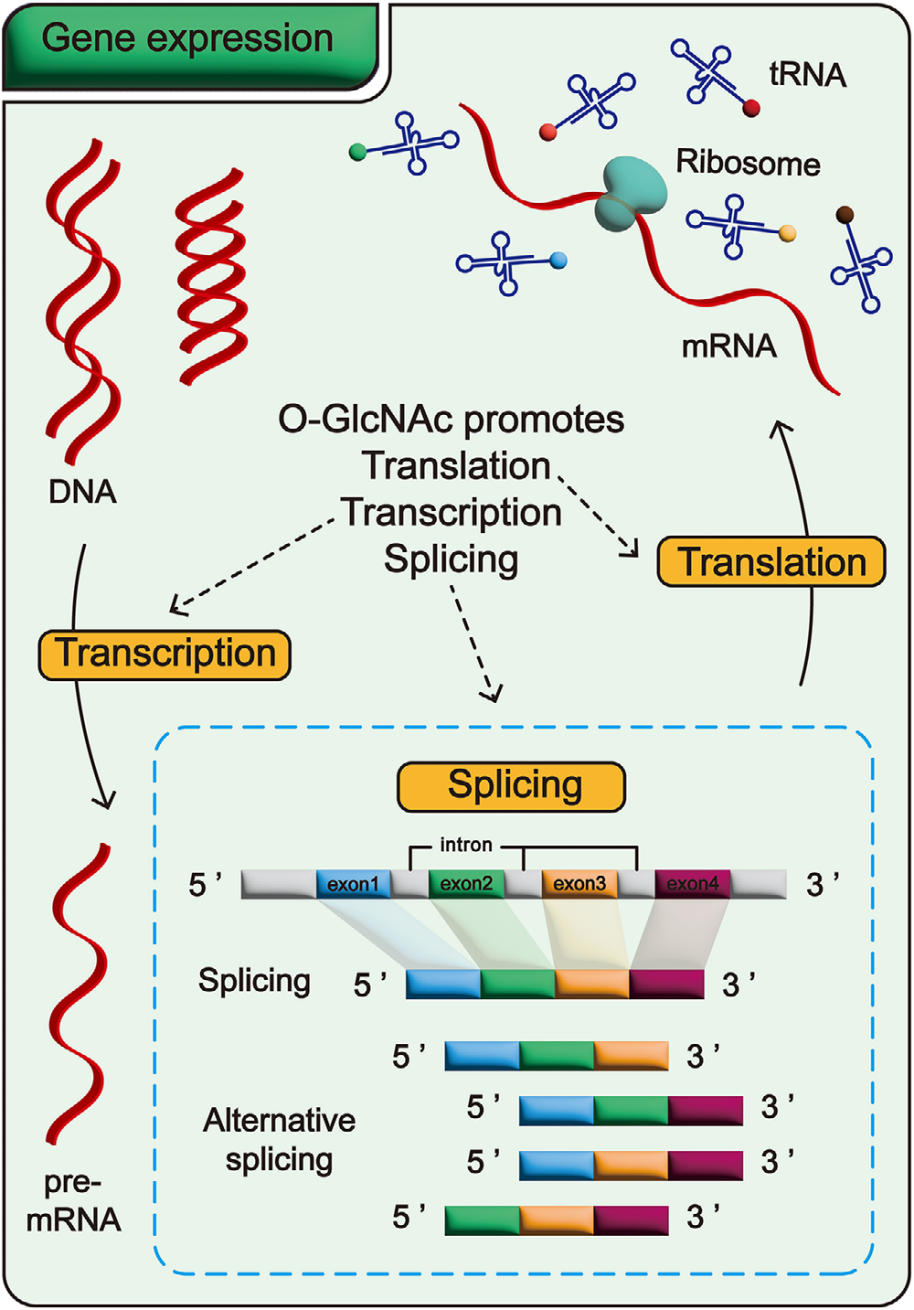
O‐GlcNAcylation promotes gene expression. In the process of gene expression, O‐GlcNAcylation plays a critical role, running through multiple stages from transcription to translation. First, transcription, as the initial step of gene expression, depends on the regulation of O‐GlcNAcylation to proceed smoothly. Next, the pre‐mRNA generated by transcription undergoes maturation processes such as splicing, which also requires the involvement of O‐GlcNAcylation. Finally, at the stage of protein translation, O‐GlcNAcylation also plays a facilitating role. Thus, OGlcNAcylation is broadly involved in and closely regulates every step of gene expression.

Gene expression, one of the most fundamental biological processes, occurs continuously in countless cells throughout an organism. In summary, O‐GlcNAcylation is involved in nearly all stages of gene expression. Key steps—including transcription, mRNA splicing, protein translation, and the functional execution of resultant proteins—are highly dependent on O‐GlcNAcylation regulation (Figure 2 and Table 1). Abnormal O‐GlcNAcylation levels can disrupt these processes, thereby impeding normal gene expression. Therefore, O‐GlcNAcylation plays an indispensable role in ensuring the smooth progression of gene expression.

**TABLE 1:** Proper protein O‐GlcNAcylation is critical for health.

O‐GlcNAcylated proteins	O‐GlcNAcylated sites	Biological events regulated by O‐GlcNAcylation	References
O‐GlcNAcylation promotes gene expression			
RNAP II	/	Promotes the assembly of PIC and facilitates transcriptional elongation and termination	[36, 37]
PIC	/	Promotes gene transcription	[38, 39]
Histone	Multiple sites	Promotes chromatin relaxation and facilitates gene transcription	[40, 41, 42, 43, 44, 45, 46, 47, 48, 49]
TBP	/	Promotes gene transcription	[50]
Topo I	/	Promotes gene transcription	[51]
NPC	/	Promotes gene transcription	[52]
HOXA1	/	Promotes gene transcription	[53, 54, 55, 56, 57, 58, 59]
Sp1	/	Promotes gene transcription	[60]
Hnf‐1	/	Promotes gene transcription	[61]
c‐Myc	/	Promotes gene transcription	[62, 63]
SRF	/	Promotes gene transcription	[64]
OGT	/	Affects mRNA splicing	[66]
OGA	/	Affects mRNA splicing	[66]
SRPK2	Ser490 Thr492 Thr498	Affects mRNA splicing	[69]
AtACINUS	/	Affects mRNA splicing	[70]
TDP‐43	/	Affects mRNA splicing	[71]
AUF1	/	Affects mRNA splicing	[72]
SGs	/	Promotes protein translation	[75, 76, 77, 78, 79]
PBs	/	Promotes protein translation	[75, 76, 77, 78, 79]
RPL5	/	Promotes ribosomal function	[80]
RPL10	/	Promotes ribosomal function	[80]
RPL11	/	Promotes ribosomal function	[80]
RPL13a	/	Promotes ribosomal function	[80]
O‐GlcNAcylation promotes the cell cycle			
CDKs	/	Promotes the G1‐to‐S transition	[82]
Cyclin D1	/	Inhibits ubiquitin–proteasome degradation	[84]
β‐Catenin	/	Promotes G0 to enter G1 phase	[85, 86]
DDX3X	Ser584	Inhibits DDX3X ubiquitin–proteasome degradation, promotes translation of cyclin E, and facilitates the G1/S transition	[87, 88]
HCF‐1	Multiple sites	Promotes G1 and M phase progression	[89]
Aurora B	/	Promotes spindle function	[91, 96]
PLK1	/	Promotes spindle function	[91, 96]
O‐GlcNAcylation promotes metabolism			
HCF‐1	Ser333	Promotes hepatic gluconeogenesis	[99]
ERRγ	Ser317 Ser319	Enhances the transcriptional activity of ERRγ and promotes the expression of gluconeogenic genes	[101]
FOXO1	/	Promotes hepatic gluconeogenesis	[102, 103, 104, 105, 106]
CRTC2	/	Promotes hepatic gluconeogenesis	[102, 103, 104, 105, 106]
PDX1	/	Promotes its nuclear localization, enhances its DNA‐binding ability, and facilitates the transcription of insulin‐related genes	[107, 108]
NeuroD1	/	Enhances the activity of the insulin gene promoter	[109, 110, 111]
O‐GlcNAcylation promotes signal transduction			
β‐catenin	Ser23 Thr40 Thr41 Thr112	Inhibits ubiquitin–proteasome degradation, promotes the activation of the Wnt signaling pathway, and drives tissue homeostasis regeneration	[121, 122, 123]
IR‐β	/	Promotes the activation process	[126]
IRS1	/	Promotes the activation process	[126]
AKT	/	Promotes the activation process	[126]
PDK1	/	Promotes the activation process	[126]
FoxO1	/	Promotes the activation process	[126]
PI3K	/	Promotes the activation process	[126]
NF‐κB	/	Enhances its interaction with downstream target genes	[132, 133]
TAB1	Ser395	Promotes downstream signal transduction	[129, 134]
INFs	/	Promotes cytokine signaling	[135]
PD‐L1	/	Promotes cytokine signaling	[136]
TGF‐β	/	Promotes cytokine signaling	[137]

### 2.2 O‐GlcNAcylation Promotes the Cell Cycle

The cell cycle, encompassing the period from the completion of one division to the next, is a fundamental biological process central to cell proliferation. It consists of interphase (G1, S, G2) and mitotic phase (M). The progression of the cell cycle is tightly regulated by various molecules, among which cyclin‐dependent kinases (CDKs) play a pivotal role. Their function is highly dependent on the maintenance of proper O‐GlcNAcylation levels [81]. Studies indicate that abnormal O‐GlcNAcylation levels significantly impair the expression stability of cyclins and genomic integrity. Elevated O‐GlcNAcylation leads to cyclin dysregulation and genomic instability, whereas inhibition of this modification disrupts the cell cycle progression—for example, by hindering the synthesis of cyclin E in G1 phase, thereby suppressing the G1‐to‐S transition [82]. It has also been reported that inhibiting OGT impedes the G2‐to‐M transition, though the underlying mechanism remains unclear [83]. Cyclin D1, a regulatory partner of CDK4/6, participates in multiple the cell cycle events and is precisely controlled through various modifications such as phosphorylation and ubiquitination. Elevated O‐GlcNAcylation inhibits its ubiquitination‐mediated degradation, enhances its stability, and thereby promotes the cell cycle progression [84]. During G0 phase, cyclin D is abundantly expressed in a β‐catenin‐dependent transcriptional manner, driving cells from quiescence into G1 phase. The stability of β‐catenin in this process also relies on O‐GlcNAcylation [85, 86]. The C‐terminal extension of RNA helicase DDX3X contains two O‐GlcNAcylation sites (Ser584 and Ser588) [87]. Modification at Ser584 inhibits DDX3X degradation via the ubiquitin–proteasome pathway, enhances its stability, promotes translation of cyclin E, and facilitates the G1/S transition [88]. The N‐ and C‐terminal cleavage products of host cell factor 1 (HCF‐1) promote G1 and M phase progression [89]. HCF‐1 itself possesses over 30 O‐GlcNAcylation sites, and its activation and stability are strongly dependent on this modification [90]. These findings collectively demonstrate that homeostasis of O‐GlcNAcylation is essential for the normal function of numerous the cell cycle‐related proteins.

Furthermore, O‐GlcNAcylation is directly involved in spindle assembly and function during mitosis and meiosis. OGT is closely associated with spindle formation, and the proper assembly of centrosomes and spindles depends on stable protein O‐GlcNAcylation [82, 91, 92, 93]. Abnormal levels of this modification disrupt methylation and acetylation patterns of spindle proteins, leading to defective spindle formation [91, 94]. Excessive O‐GlcNAcylation also causes structural abnormalities: during M phase, overexpression promotes the formation of smaller, disorganized compact chromatid structures [95], inhibits CDK1‐mediated transcriptional activation of phosphatase CDC25c, and impairs phosphorylation of CDK1 downstream substrates, thereby disrupting mitotic spindle assembly [91]. Elevated O‐GlcNAcylation can also alter the phosphorylation of spindle proteins through mitotic kinases Aurora B and Polo‐like kinase 1 (PLK1), ultimately impairing spindle function [91, 96]. Under OGA knockdown conditions, sustained high O‐GlcNAcylation suppresses CDK1 phosphorylation, hinders cyclin B/CDK1 complex formation [97], and causes mislocalization of the spindle component Ewing sarcoma breakpoint region 1 protein, resulting in multipolar spindle structures [98]. Both elevated and reduced O‐GlcNAcylation levels significantly disrupt normal spindle structure and function, indicating that dynamic balance of this modification is crucial for maintaining spindle integrity and ensuring orderly the cell cycle progression.

**FIGURE 3:**
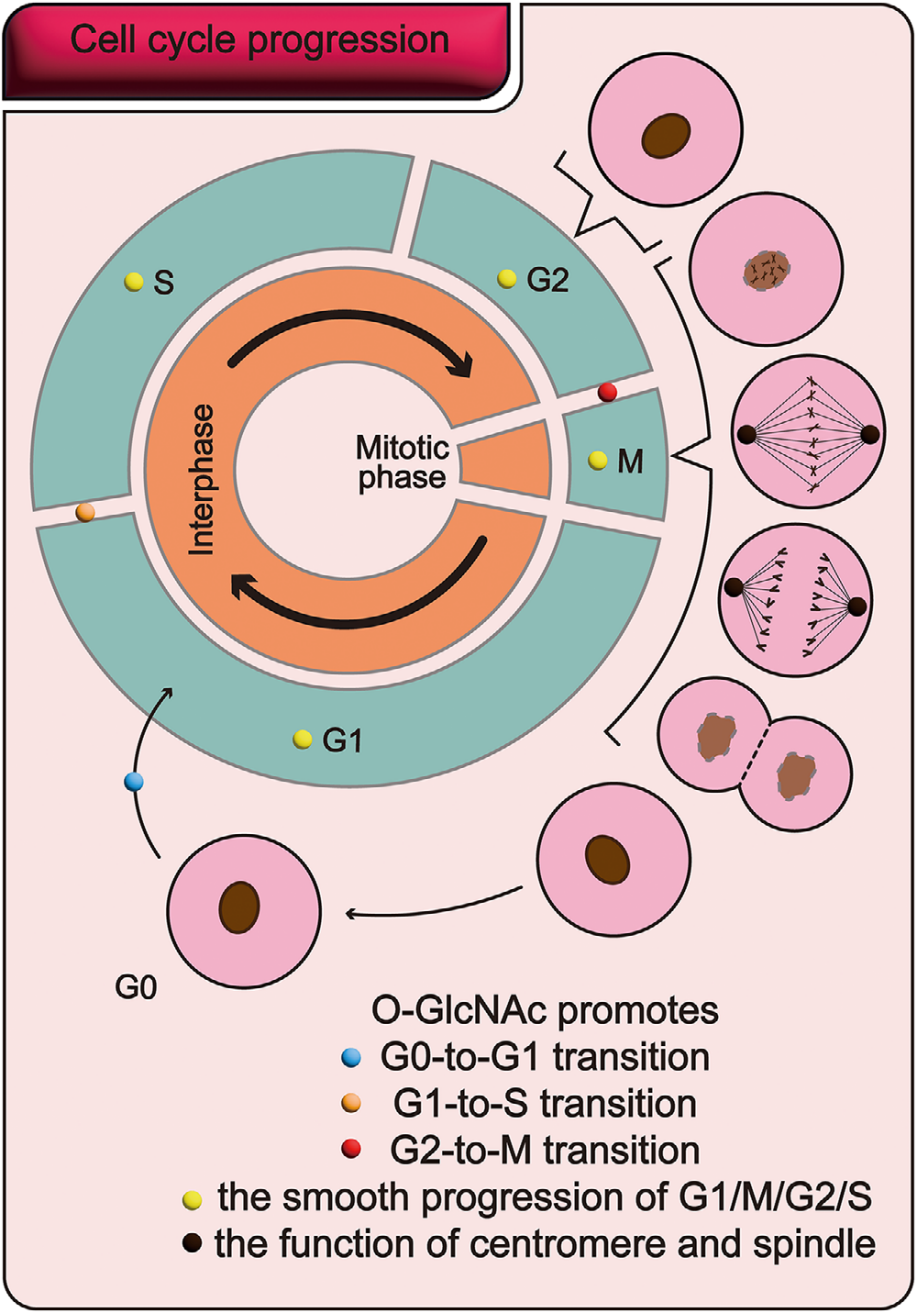
O‐GlcNAcylation promotes the cell cycle. O‐GlcNAcylation is a crucial regulator of the orderly progression of the cell cycle. First, it directly modulates the progression of various phases of the cell cycle, serving as an essential factor for the proper advancement of interphase (G1, S, G2) and mitosis (M). It also facilitates key transitions such as G0‐to‐G1, G1‐to‐S, and G2‐to‐M. Additionally, by maintaining the structural and functional stability of centrosomes and spindles, it ensures the smooth execution of cell division. Thus, O‐GlcNAcylation is an indispensable regulatory factor for the orderly progression of all phases and stages of the cell cycle.

The cell cycle, as the core process of cell proliferation, is closely related to the growth and development of organisms. Studies have shown that O‐GlcNAcylation is extensively involved in regulating the cell cycle, spanning the G0, G1, S, G2, and M phases (Figure 3). A normal and stable level of O‐GlcNAcylation is crucial for maintaining the function of the cell cycle‐related proteins and catalytic enzymes (Table 1). Furthermore, it plays a key supporting role in centrosome duplication and proper spindle assembly. Conversely, abnormal O‐GlcNAcylation can significantly disrupt the cell cycle, leading to dysregulated phase transitions and disturbances in various cycle‐related molecular events. Therefore, maintaining the dynamic balance of O‐GlcNAcylation is essential for ensuring the orderly progression of the cell cycle.

### 2.3 O‐GlcNAcylation Promotes Metabolism

O‐GlcNAcylation plays a critical regulatory role in the process of gluconeogenesis, a metabolic pathway through which the body converts noncarbohydrate substrates into glucose or glycogen. This process is essential for maintaining blood glucose homeostasis and energy supply during states such as starvation or stress. Studies have shown that under physiological stress, OGT collaborates with HCF‐1 to sense intracellular glucose availability and catalyze the O‐GlcNAcylation of Ser333 on the transcriptional coactivator PGC‐1α. This modification significantly enhances both the activation and protein stability of PGC‐1α, thereby promoting hepatic gluconeogenesis [99]. Estrogen‐related receptor γ (ERRγ), a key positive regulator of liver gluconeogenesis, is also precisely modulated by O‐GlcNAcylation [100]. As an important substrate of OGT, ERRγ can be O‐GlcNAcylated at Ser317 and Ser319. This modification enhances the transcriptional activity of ERRγ, leading to increased expression of downstream gluconeogenic genes and further driving hepatic glucose production [101]. Additionally, O‐GlcNAcylation can upregulate the expression of gluconeogenic genes by modifying transcription factors such as FOXO1 and CRTC2, thereby promoting hepatic gluconeogenesis through multiple pathways [102, 103, 104, 105, 106]. Thus, in response to metabolic demand for gluconeogenesis, O‐GlcNAcylation stabilizes and activates key regulatory proteins through various mechanisms, enhances the transcriptional expression of gluconeogenesis‐related genes, and ultimately facilitates the conversion of noncarbohydrate precursors into glucose, providing essential energy support for the organism.

O‐GlcNAcylation, as a key glycosylation modification, plays a central regulatory role in insulin synthesis and secretion. Its modification level is directly influenced by glucose metabolism, thereby participating in the critical mechanisms regulating insulin secretion. Studies have shown that inhibiting GFAT, the rate‐limiting enzyme of HBP, leads to a significant decrease in O‐GlcNAcylation levels and severely suppresses insulin secretion, fully demonstrating the indispensability of this modification in insulin secretion. At the molecular level, O‐GlcNAcylation functions by modifying multiple key transcription factors. For example, O‐GlcNAcylation of the pancreatic transcription factor PDX1 promotes its nuclear localization, enhances its DNA‐binding ability, and thereby facilitates the transcription of insulin‐related genes [107, 108]. In pancreatic‐derived MIN6 cells, high glucose conditions significantly increase the O‐GlcNAcylation level of PDX1, correspondingly enhancing insulin gene expression and secretion [108]. Similarly, the transcription factor NeuroD1 is also modified by O‐GlcNAcylation. Modified NeuroD1 markedly enhances the activity of the insulin gene promoter [109, 110, 111]. Under high glucose conditions, O‐GlcNAcylated NeuroD1 tends to localize in the nucleus with improved promoter‐binding capacity, effectively promoting insulin gene transcription and protein synthesis [110, 111]. Thus, O‐GlcNAcylation modifies key transcription factors such as PDX1 and NeuroD1, facilitating their nuclear translocation, strengthening their DNA or promoter binding abilities, and synergistically upregulating the transcription of insulin‐related genes, ultimately promoting insulin biosynthesis and secretion.

**FIGURE 4:**
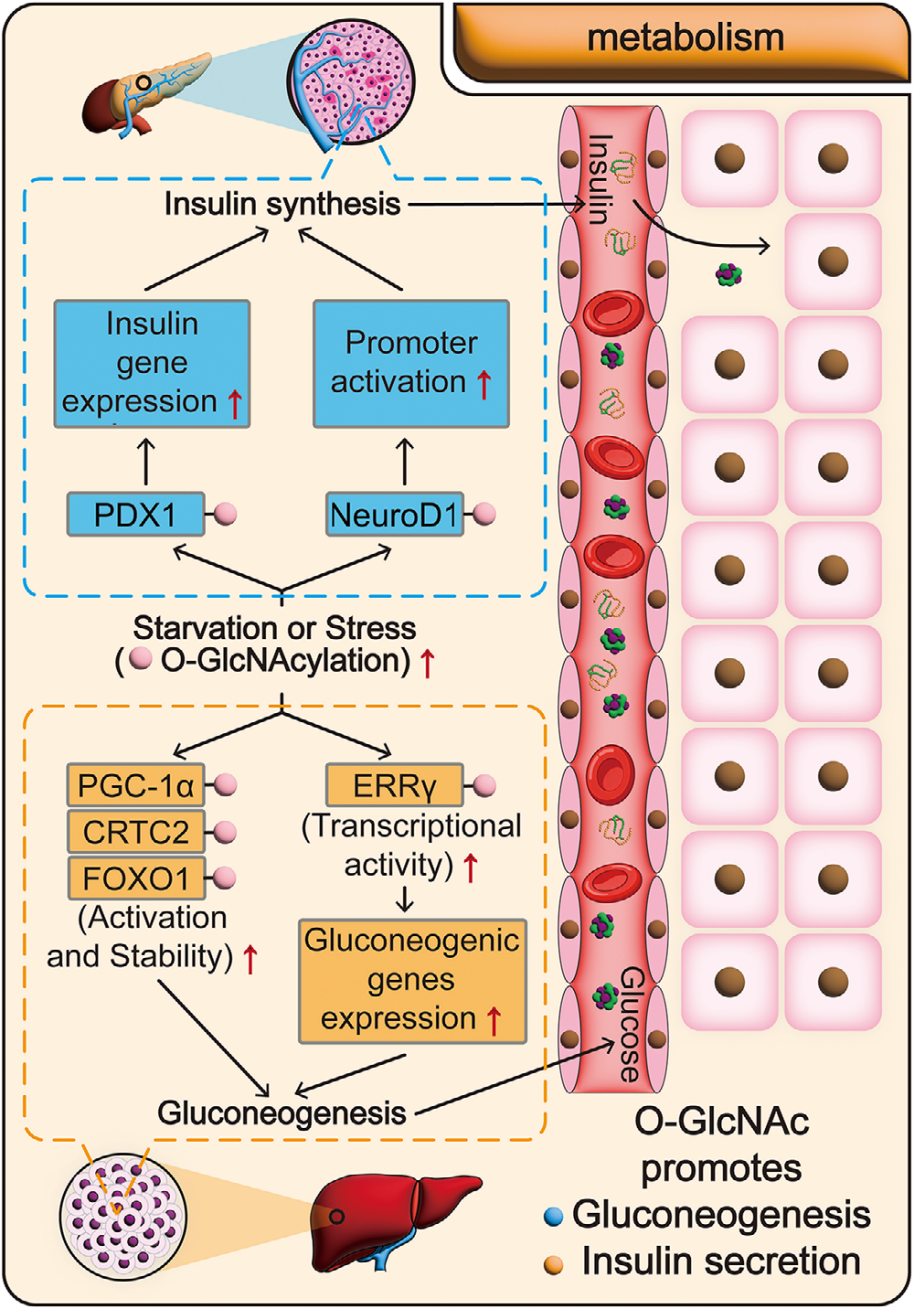
O‐GlcNAcylation promotes metabolism. Under extreme hunger or stress, the body's protein O‐GlcNAcylation levels rise significantly, thereby enhancing metabolic processes. First, O‐GlcNAcylation upregulates the expression of gluconeogenesis‐related genes in liver cells, promoting gluconeogenesis and increasing blood glucose levels. Second, OGlcNAcylation stimulates pancreatic β‐cells to synthesize and release insulin, which facilitates glucose uptake and utilization by body cells, thereby lowering blood glucose. Thus, O‐GlcNAcylation serves as a crucial mechanism for energy security under abnormal physiological conditions, maintaining internal homeostasis and overall health.

Cellular metabolism serves as the central hub for energy supply in organisms, directly maintaining energy homeostasis and metabolic stability. Among these processes, O‐GlcNAcylation, as an important posttranslational modification, is closely linked to glucose metabolism. Research has demonstrated that under conditions of hunger or stress, O‐GlcNAcylation enhances insulin secretion and gluconeogenesis, thereby maintaining blood glucose levels and ensuring a continuous energy supply for normal physiological activities. This highlights its crucial role in sustaining health (Figure 4 and Table 1). However, current research on the functions of O‐GlcNAcylation in metabolic pathways beyond glucose metabolism—such as lipid and amino acid metabolism—remains limited. Its regulatory mechanisms and physiological significance require further in‐depth exploration.

### 2.4 O‐GlcNAcylation Promotes Signal Transduction

Cells sense and respond to changing external conditions by detecting physicochemical cues from their environment. This process, known as signal transduction, involves converting extracellular signals into intracellular responses [112, 113]. As a key posttranslational modification, O‐GlcNAcylation regulates the activity and stability of numerous signaling molecules. Through this modification, it facilitates the activation and maintenance of downstream signaling pathways.

O‐GlcNAcylation plays a crucial role in regulating G protein‐coupled receptors (GPCRs) signal transduction. As the largest family of signaling receptors in the human body, GPCRs are widely involved in various biological processes, enabling cells to respond to external stimuli [114, 115]. Among them, the Wnt signaling pathway is a typical GPCR‐mediated pathway that participates in key biological processes such as tissue homeostasis and regeneration, stem cell proliferation, and differentiation [116, 117]. This pathway is precisely regulated by O‐GlcNAcylation [118]. Within the Wnt pathway, β‐catenin serves as a core transcription factor. Under resting cellular conditions, β‐catenin is inactivated through continuous phosphorylation and degraded via ubiquitination, preventing it from performing transcriptional functions [119, 120, 121]. However, during active cell proliferation, OGT catalyzes the O‐GlcNAcylation of multiple amino acid residues on β‐catenin, including Ser23, Thr40, Thr41, and Thr112 [122]. This modification further promotes the de‐phosphorylation and de‐ubiquitination of β‐catenin, significantly enhancing its activation level and protein stability. Activated β‐catenin initiates downstream signaling cascades, promotes the activation of the Wnt signaling pathway, and effectively drives tissue homeostasis regeneration and other related biological processes [121, 122, 123]. Thus, by positively regulating the stability and activity of β‐catenin, O‐GlcNAcylation plays a key role in GPCR/Wnt signal transduction.

O‐GlcNAcylation plays a critical role in promoting receptor tyrosine kinases (RTKs) signal transduction. An important class within the RTKs protein family is growth factor receptors, which regulate multiple signaling pathways, such as the protein kinase B (AKT) pathway that controls cell growth and proliferation [124], and the insulin signaling pathway that regulates metabolism and energy storage [125]. Notably, in both the AKT and insulin pathways, several key signaling molecules—including IR‐β, IRS1, AKT, pyruvate dehydrogenase kinase (PDK)1, FoxO1, and PI3K—are direct targets of OGT. Their activation or inactivation processes rely on O‐GlcNAcylation [126]. Furthermore, O‐GlcNAcylation also modulates the MAPK signaling pathway, a classic downstream pathway of RTKs that regulates cell proliferation, differentiation, and apoptosis [127, 128]. Although there are currently few reports of OGT directly modifying MAPK components, existing evidence suggests that O‐GlcNAcylation can enhance interactions between upstream and downstream signaling molecules in the MAPK pathway through crosstalk with phosphorylation modifications, thereby effectively promoting the activation and propagation of this signaling pathway [129].

O‐GlcNAcylation plays a crucial regulatory role in cytokine signal transduction. Various cytokines, such as tumor necrosis factor α (TNFα) and interleukins (ILs), are primarily involved in regulating inflammatory and immune cell processes [130], and O‐GlcNAcylation serves as a key modification in the regulation of these cellular functions [131]. For instance, O‐GlcNAcylation significantly influences biological processes such as cell proliferation and apoptosis by activating nuclear factor κB (NF‐κB) in response to external stimuli [132]. NF‐κB typically exists as a dimer composed of p65 and p50 subunits, with the activity and function of p65 being highly regulated by O‐GlcNAcylation. This modification promotes the nuclear translocation of NF‐κB and enhances its interaction with downstream target genes, thereby effectively initiating related signaling pathways [133]. Additionally, TAB1, an upstream regulatory protein of NF‐κB, is also modulated by O‐GlcNAcylation. Liquid chromatography–tandem mass spectrometry analysis has identified Ser395 of TAB1 as a key site for O‐GlcNAcylation. Mutation at this site significantly inhibits TAB1 activation, and further studies confirm that in ILs signaling pathways, O‐GlcNAcylation actively promotes downstream signal transduction by enhancing TAB1 activity [129, 134]. Beyond TNFα and ILs, the activation processes of other cytokines, such as interferons [135], programmed death‐ligand 1 (PD‐L1) [136], and transforming growth factor β (TGF‐β) [137], also widely depend on O‐GlcNAcylation. These findings collectively demonstrate that O‐GlcNAcylation plays a key positive regulatory role in multiple cytokines signaling pathways, significantly facilitating the smooth progression of inflammatory and immune responses.

**FIGURE 5:**
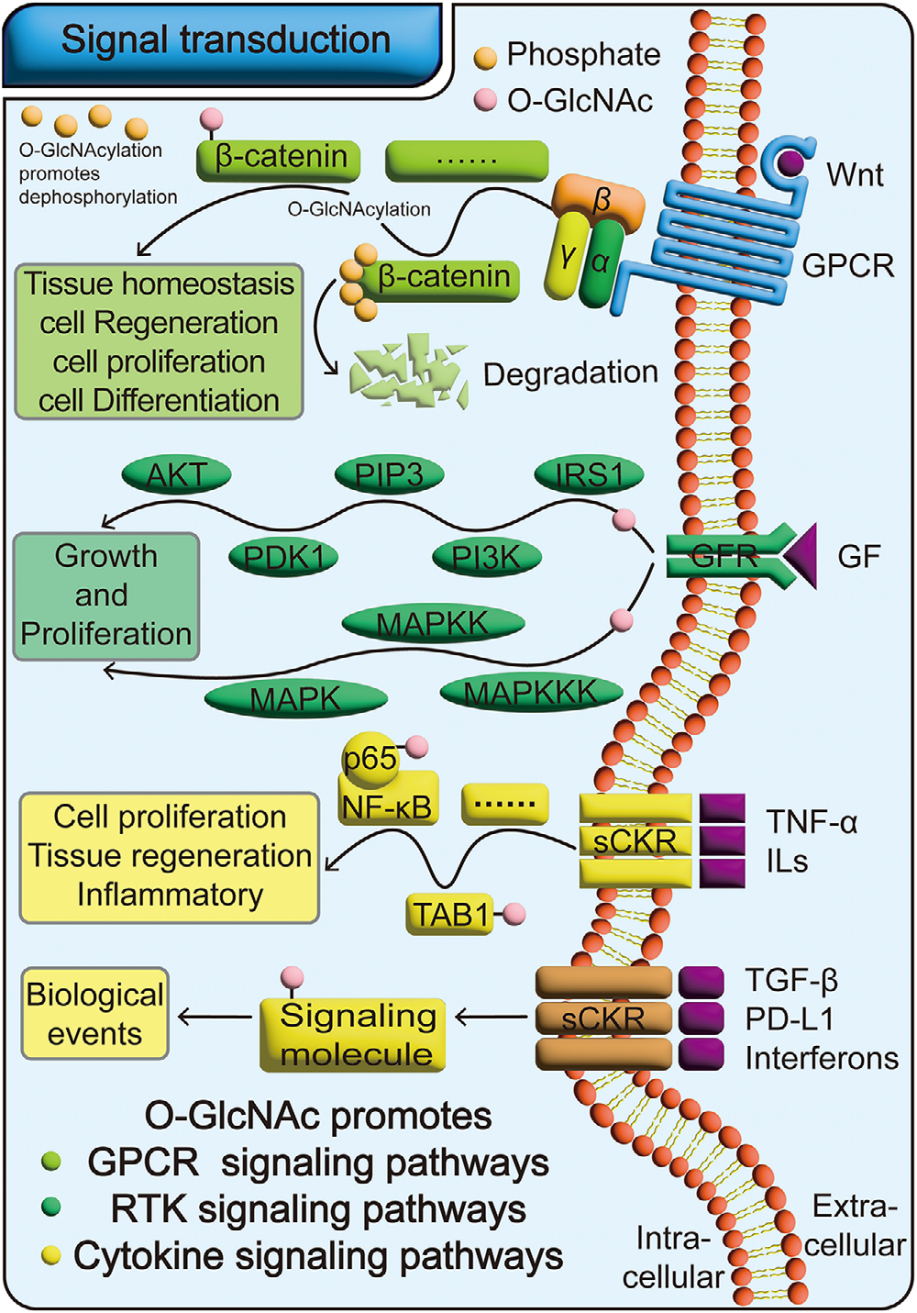
O‐GlcNAcylation promotes signal transduction. O‐GlcNAcylation is widely involved in regulating various cellular signal transduction, including GPCRs signaling, RTKs signaling, and cytokine receptor signaling. Specifically, it modifies upstream and downstream signaling molecules and enhances their activation, thereby facilitating the efficient transmission of biological information within the pathways. Thus, this modification plays a crucial role in maintaining the openness and proper functioning of signaling pathways.

In summary, classical signaling pathways such as GPCR, RTK, and cytokine pathways form critical regulatory networks that ensure the proper execution of various biological processes. Dysregulation of these pathways can lead to disruptions in multiple biological functions. Notably, key molecules within these pathways—including receptors, kinases, and transcription factors—are highly dependent on O‐GlcNAcylation to maintain their stability and activation (Table 1). Therefore, during cellular responses to external stimuli, O‐GlcNAcylation not only significantly influences the activation and deactivation of related signaling pathways through precise regulation of signaling molecules but also provides an indispensable molecular foundation for efficient and orderly signal transduction (Figure 5).

## 3 Abnormal O‐GlcNAcylation Contributes to Disease Pathogenesis

Normal O‐GlcNAcylation plays a crucial role in maintaining overall health, actively regulating various biological processes such as gene expression, the cell cycle, metabolism, and signal transduction. It serves as an essential molecular foundation for sustaining internal homeostasis. However, in the development and progression of numerous diseases, dysregulation of O‐GlcNAcylation often serves as a key driver of pathogenesis. This involves various pathological conditions, including neurodegenerative diseases, CVDs, cancers, immune‐related diseases. Therefore, this section will systematically elaborate on the specific roles of O‐GlcNAcylation abnormalities in these diseases and provide an in‐depth analysis of the associated biological processes and molecular mechanisms driving disease development.

### 3.1 Abnormal O‐GlcNAcylation Contributes to the Pathogenesis of Neurodegenerative Diseases

Neurodegenerative diseases are a group of progressive neurological disorders characterized primarily by cognitive and physical dysfunction, with the core pathological mechanism lying in the gradual loss of neuronal structure and function. Neurons, as the fundamental units of the nervous system, rely heavily on the precise regulation of various posttranslational modifications to maintain normal function. Among these, O‐GlcNAcylation—a key dynamic modification—is highly abundant in the brain, particularly at synaptic sites. In functional synapses, both OGT and OGA are actively expressed and work together to maintain the dynamic balance of O‐GlcNAcylation at nerve terminals [138]. Numerous proteins closely associated with neuronal structure and function, such as CaMKIV, GluA2, synapsin I, synaptopodin, piccolo, bassoon, cAMP response element‐binding protein, and calcium/calmodulin‐dependent protein kinase II (CaMKII), are precisely regulated by O‐GlcNAcylation [139, 140, 141, 142, 143, 144]. Therefore, O‐GlcNAcylation homeostasis is essential for maintaining normal neuronal physiological function. Disruption of this modification can lead to structural and functional abnormalities in neurons, thereby contributing to the onset and progression of diseases. In recent years, growing evidence has shown that aberrant O‐GlcNAcylation plays a significant role in triggering and promoting various neurodegenerative diseases, including PD, HD, AD, and ALS. The following content will elaborate on the specific mechanisms through which abnormal O‐GlcNAcylation contributes to neurodegenerative diseases.

Abnormal O‐GlcNAcylation levels are closely associated with the onset and progression of AD. As one of the most common neurodegenerative diseases, AD is clinically characterized by memory impairment, mood fluctuations, and cognitive decline [145]. Multiple studies have shown that O‐GlcNAcylation levels in the brain tissues of AD patients are 22–50% lower than those in healthy individuals, suggesting that reduced O‐GlcNAcylation may contribute to AD pathology [146, 147, 148, 149, 150]. At the molecular level, aberrant O‐GlcNAcylation promotes AD progression primarily by affecting the metabolism of Tau protein and β‐amyloid (Aβ). Tau is a microtubule‐associated protein critical for maintaining neuronal cytoskeletal stability. Its activity is regulated by phosphorylation and it contains multiple O‐GlcNAcylation sites, such as Thr123, Ser208, Ser400, Ser409, Ser412, and Ser413 [151]. In AD, Tau undergoes abnormal hyperphosphorylation and aggregates into neurofibrillary tangles (NFTs), which is one of the core pathological features [152, 153]. Cellular and animal studies have demonstrated that elevating O‐GlcNAcylation levels with the OGA inhibitor thiamet‐G significantly suppresses phosphorylation of Tau at sites including Ser396, Thr231, Ser202, and Thr205, thereby inhibiting its hyperphosphorylation and aggregation [154, 155]. This indicates that O‐GlcNAcylation can counteract tauopathy by antagonizing Tau phosphorylation, and its reduction may facilitate AD progression. In addition, Aβ is a peptide fragment produced through sequential cleavage of the conserved type I transmembrane protein APP by α [156], β [157], and γ‐secretase complexes [158, 159], and it accumulates abnormally in the brains of AD patients [160]. Studies have confirmed that O‐GlcNAcylation also regulates Aβ production [161]. In AD model mice overexpressing Aβ, treatment with thiamet‐G significantly reduces Aβ levels [162]. Further mechanistic research reveals that O‐GlcNAcylation modifies the Ser708 residue of the γ‐secretase subunit nicastrin (NCT), thereby inhibiting γ‐secretase activity and reducing Aβ generation [163]. This again suggests that elevated O‐GlcNAcylation suppresses Aβ‐related pathology, while its downregulation promotes amyloid disease. Beyond regulating Tau and Aβ, O‐GlcNAcylation is involved in AD progression through other biological processes such as neuroinflammation and insulin signaling. For example, loss of OGT promotes astrocyte activation and inflammatory responses, leading to cognitive impairment in mice; restoring O‐GlcNAcylation alleviates inflammation and improves cognitive function. Mechanistically, OGT catalyzes O‐GlcNAcylation at Ser384 of the NF‐κB p65 subunit in glial cells, inhibiting NF‐κB signaling and exerting anti‐inflammatory effects. Conversely, its deficiency promotes NF‐κB activation and enhances neuroinflammation [164]. In AD, decreased O‐GlcNAcylation leads to overactivation of the NF‐κB pathway, exacerbating neuroinflammation and accelerating cognitive decline. Additionally, cerebral insulin resistance is another key factor in AD development [165, 166]. Insulin receptor substrate‐1 (IRS‐1) is a critical molecule in the insulin signaling pathway, and phosphorylation at its Ser307 site is closely linked to insulin resistance. Multiple Ser/Thr residues of IRS‐1 can also undergo O‐GlcNAcylation [167, 168]. During AD progression, the decline in O‐GlcNAcylation occurs earlier than IRS‐1 phosphorylation. Since these two modifications may compete for the same or adjacent sites, reduced O‐GlcNAcylation may lift the inhibition on phosphorylation, thereby promoting IRS‐1 phosphorylation, insulin resistance, and further AD development [168]. In summary, abnormally low O‐GlcNAcylation levels significantly promote the initiation and progression of AD by regulating key events including Tau phosphorylation, Aβ production, neuroinflammatory responses, and insulin signaling. Restoring O‐GlcNAcylation levels may represent a potential therapeutic strategy for AD intervention.

PD is the second most common neurodegenerative disease after AD. Its primary clinical features include bradykinesia, rigidity, tremor, and neuropsychiatric symptoms [169]. The characteristic neuropathological hallmarks of PD include the loss of dopaminergic neurons in the substantia nigra and the formation of Lewy bodies composed of abnormally aggregated α‐synuclein [170]. Recent studies have shown that decreased levels of O‐GlcNAcylation are closely associated with these pathological processes [169, 171, 172, 173]. In the development of PD, abnormal phosphorylation of α‐synuclein at Ser87 and Ser129 promotes its aggregation [172] and widespread distribution in presynaptic terminals and synaptic vesicles, interfering with critical functions such as vesicle stability, transport, and neurotransmitter release [174, 175]. α‐synuclein can be modified by O‐GlcNAcylation [172, 173]: cellular experiments have confirmed that O‐GlcNAcylation at Thr72, Thr75, and Thr81 inhibits phosphorylation at Ser87 and Ser129, thereby reducing α‐synuclein aggregation and its neurotoxicity and slowing PD progression [176, 177, 178]. Beyond its regulatory role on α‐synuclein, reduced O‐GlcNAcylation levels also directly impair dopaminergic neuron function [171]. Studies have shown that specific knockout of OGT in dopaminergic neurons leads to neuronal loss, whereas increasing O‐GlcNAcylation supports normal neuronal function and enhances synaptic transmission [179]. In PD mouse models, elevating O‐GlcNAcylation levels by inhibiting OGA significantly alleviates pathological damage in dopaminergic neurons, improves dopamine release deficits and motor dysfunction, and reduces α‐synuclein aggregation [179]. In summary, upregulation of O‐GlcNAcylation is neuroprotective event, while its reduction promotes key pathological processes such as abnormal α‐synuclein aggregation and dopaminergic neuron loss, thereby exacerbating the onset and progression of PD. Therefore, targeting O‐GlcNAcylation may represent a potential therapeutic strategy for PD.

Abnormal O‐GlcNAcylation plays a significant role in the onset and progression of HD. HD is a rare neurodegenerative disease characterized primarily by cognitive and motor dysfunction [180, 181]. Its pathogenesis stems from mutations in the huntingtin gene *HTT*, leading to misfolding of the encoded huntingtin protein (HTT), which gradually forms oligomers, polymers, and pathogenic mutant HTT fibrils (mHTT fibrils). These abnormal protein aggregates further disrupt key biological processes such as neuronal synaptic transmission, mitochondrial function, and gene transcription, ultimately causing the disease [182, 183]. The structure and function of the HTT protein and its interacting protein HIP1R are regulated by O‐GlcNAcylation [171, 184, 185]. Studies have shown that coexpression of mutant HTT and OGA in mouse neural cells results in reduced O‐GlcNAcylation levels, accompanied by a significant decrease in misfolded HTT proteins and a marked reduction in cell death caused by abnormal mHTT aggregation [185]. This clearly indicates that O‐GlcNAcylation levels are negatively correlated with HD progression, and its elevated expression promotes HD development. Besides, disrupted nucleocytoplasmic transport and distorted nuclear membranes are well‐established pathological features in HD model mice. The NPC, a critical channel for nucleocytoplasmic transport, relies on the proper function of NUPs, whose structural stability and selective permeability are highly dependent on O‐GlcNAcylation [186, 187]. Experiments revealed that treating HD mice with OGA inhibitors increased O‐GlcNAcylation levels and significantly restored nucleocytoplasmic transport function, suggesting that insufficient O‐GlcNAcylation promotes HD pathological progression by impairing NUP function and nucleocytoplasmic transport [186]. In summary, the role of O‐GlcNAcylation in HD is complex and context‐dependent. On one hand, elevated O‐GlcNAcylation promotes the abnormal aggregation of HTT proteins. On the other hand, reduced O‐GlcNAcylation impairs the function of the NPC. Both abnormalities significantly accelerate HD progression, indicating that an imbalance in O‐GlcNAcylation levels—whether increased or decreased—plays a critical role in the development and progression of HD.

Abnormal O‐GlcNAcylation levels play a significant role in the pathological progression of ALS. ALS is a neurodegenerative disease characterized by dysfunction of both upper and lower motor neurons, with clinical manifestations including motor impairment, dysarthria, dysphagia, and behavioral or cognitive abnormalities [188, 189, 190, 191, 192]. One of the hallmark pathological features of ALS is the formation of axonal spheroids composed of neurofilament (NF) proteins [193]. NF proteins are coregulated by O‐GlcNAcylation and phosphorylation, which exhibit crosstalk [194, 195, 196]. In ALS, NF proteins become hyperphosphorylated and form abnormal aggregates [197, 198], while O‐GlcNAcylation levels are significantly reduced in ALS mouse models [199]. This suggests that diminished O‐GlcNAcylation allows phosphorylation to dominate, promoting NF aggregation and accelerating disease progression. Additionally, TDP‐43, a critical RNA/DNA‐binding protein involved in transcriptional regulation and stability maintenance, forms toxic aggregates that contribute to ALS pathogenesis [200, 201, 202]. O‐GlcNAcylation occurs at Thr199 and Thr233 residues of TDP‐43. Studies show that co‐overexpression of OGT and TDP‐43 in SH‐SY5Y neuroblastoma cells effectively suppresses TDP‐43 aggregation and cytotoxicity, indicating a protective role of O‐GlcNAcylation [203]. Reduced O‐GlcNAcylation promotes TDP‐43 aggregation and facilitates ALS development. Moreover, elevated oxidative stress is another key mechanism driving motor neuron degeneration. Nonselenocysteine‐containing phospholipid hydroperoxide glutathione peroxidase (NPGPx), an oxidative stress sensor, is closely associated with ALS. NPGPx knockout mice exhibit ALS‐like phenotypes, including reactive oxygen species (ROS) accumulation and motor neuron death, accompanied by a global reduction in O‐GlcNAcylation. Treatment with an OGA inhibitor in ALS mice significantly alleviates motor neuron death and ROS accumulation, indicating that decreased O‐GlcNAcylation impairs NPGPx function and exacerbates oxidative stress and neuronal death [204]. In summary, normal O‐GlcNAcylation levels may have a protective effect in ALS, whereas its downregulation promotes multiple key pathological processes—including NF assembly, TDP‐43 aggregation, and NPGPx‐mediated oxidative stress—thereby driving ALS progression. Therefore, elevating O‐GlcNAcylation levels may represent a novel therapeutic strategy for ALS.

Abnormal O‐GlcNAcylation levels have been widely recognized as a key molecular hallmark of neurodegenerative diseases. In various types of neurodegenerative diseases, dysregulated O‐GlcNAcylation significantly contributes to disease initiation and progression by impairing the function of critical proteins (Table 2). In AD, abnormally low O‐GlcNAcylation of Tau and Aβ leads to their dysfunction, representing a major pathogenic mechanism. Similarly, in PD, reduced O‐GlcNAcylation not only disrupts normal dopaminergic neuronal function but also exacerbates the abnormal aggregation and pathology of α‐synuclein. In ALS, diminished O‐GlcNAcylation of proteins such as NF, TDP‐43, and NPGPx is closely associated with disease development, resulting in decreased protein stability, increased aggregation propensity, and accelerated neurodegeneration. HD presents a slightly different scenario: hyper‐O‐GlcNAcylation of mHTT protein combined with hypo‐O‐GlcNAcylation of NUP constitutes a critical biological event driving pathological progression. Thus, abnormally low O‐GlcNAcylation plays a central role in the progression of most neurodegenerative diseases, while in certain disorders like HD, abnormally high O‐GlcNAcylation of specific proteins also serves as a non‐negligible pathogenic factor. The balance of this modification is essential for maintaining neuronal homeostasis, and its dysregulation has emerged as a common mechanism underlying neurodegenerative diseases.

**TABLE 2:** Dysregulation of protein O‐GlcNAcylation promotes disease progression.

O‐GlcNAcylated proteins	Changes in O‐GlcNAcylation levels	O‐GlcNAcylated sites	Biological events regulated by O‐GlcNAc	References
Abnormal O‐GlcNAcylation contributes to the pathogenesis of neurodegenerative diseases				
Tau	Downregulation	Thr123 Ser208 Ser400 Ser409 Ser412 Ser413	Promotes abnormal aggregation of Tau protein to form NFTs and facilitates AD progression	[151]
Aβ	Downregulation	/	Promotes abnormal aggregation of Aβ and accelerates AD progression	[161]
NCT	Downregulation	Ser708	Promotes abnormal aggregation of Aβ and accelerates AD progression	[163]
p65	Downregulation	Ser384	Promotes astrocyte activation and inflammatory responses	[164]
IRS‐1	Downregulation	/	Promotes IRS‐1 phosphorylation, insulin resistance, and further AD development	[167, 168]
α‐Synuclein	Downregulation	Thr72 Thr75 Thr81	Promotes the aggregation and neurotoxicity of α‐synuclein, thereby contributing to the progression of PD	[176, 177, 178]
HTT	Upregulation	/	Promotes the abnormal aggregation of HTT	[171, 184, 185]
HIP1R	Upregulation	/	Promotes the abnormal aggregation of HTT	[171, 184, 185]
NUPs	Downregulation	/	Impairs NUP function and nucleocytoplasmic transport	[186, 187]
NF	Downregulation	/	Promotes the phosphorylation and abnormal aggregation of the NF protein	[199]
TDP‐43	Downregulation	Thr199 Thr233	Promotes TDP‐43 protein abnormal aggregation and cytotoxicity	[203]
NPGPx	Downregulation	/	Impairs NPGPx function and exacerbates oxidative stress and neuronal death	[204]
Abnormal O‐GlcNAcylation drives cancer progression				
RBM14	Upregulation	Ser521	Enhances RBM14 transcriptional activity and promotes the proliferation and metastasis of lung cancer cells	[211, 212, 213]
NRF2	Upregulation	Ser103	Promotes the stabilization and activation of NRF2	[214]
c‐Myc	Upregulation	/	Promotes tumor invasion and metastasis	[217]
SAM68	Upregulation	/	Promotes tumor invasion and metastasis	[218]
YEATS2	Upregulation	/	Promotes tumor invasion and metastasis	[219]
PRPS1	Upregulation	Ser83 Thr166	Promotes nucleotide synthesis, supporting rapid proliferation, tumor growth, and drug resistance in lung cancer cells	[221]
SMAD4	Upregulation	/	Activates the TGF‐β signaling pathway to induce epithelial–mesenchymal transition	[226]
p‐JNK	Upregulation	/	Facilitates JNK/c‐Jun/AP‐1 signal transduction, exacerbate endoplasmic reticulum stress	[231, 232]
p‐c‐Jun	Upregulation	/	Facilitates JNK/c‐Jun/AP‐1 signal transduction, exacerbating endoplasmic reticulum stress	[231, 232]
AP‐1	Upregulation	/	Facilitates JNK/c‐Jun/AP‐1 signal transduction, exacerbating endoplasmic reticulum stress	[231, 232]
p‐IKKa/IKKb	Upregulation	/	Enhances the DNA‐binding activity of NF‐κB, promoting palmitic acid metabolism	[230]
p‐p65	Upregulation	/	Enhances the DNA‐binding activity of NF‐κB, promoting palmitic acid metabolism	[230]
p‐p50	Upregulation	/	Enhances the DNA‐binding activity of NF‐κB, promoting palmitic acid metabolism	[230]
RAF1	Upregulation	/	Inhibits ubiquitination‐mediated degradation of RAF1. Facilitates liver cancer proliferation and EMT	[234, 235]
YAP	Upregulation	Thr241	Promotes the stabilization and activation of YAP; hepatocarcnogenesis.	[238, 239]
TS	Upregulation	Thr251 Thr306	Inhibits TS proteasomal degradation; Inhibits TS proteasomal degradation	[243]
YY1	Upregulation	Thr236	Activates the expression of oncogenes SLC22A15 and AANAT; facilitates tumorigenesis	[245]
YTHDF1	Upregulation	Ser196 Ser197 Ser198	Promotes c‐Myc oncogene expression and tumor cell proliferation	[246, 247, 248]
CSNK2A1	Upregulation	/	Upregulates the expression of oncogene CSNK2A1	[249]
c‐Myc	Upregulation	Ser415	Promotes the Warburg effect and tumor cell proliferation	[250, 251]
FASN	Upregulation	/	Accelerates tumor growth and metastasis	[254]
ITGA5	Upregulation	/	Promotes tumor cell adhesion, invasion, and EMT	[255, 256, 257, 258, 259]
EZH2	Upregulation	/	Promotes tumor cell metastasis	[260]
MORC2	Upregulation	Thr556	Promotes the expression of TGF‐β1 downstream target oncogenes CTGF and SNAIL. Promotes the invasion and metastasis of tumor cells	[263]
Histone H2A	Upregulation	Ser40	Inhibits the histone demethylase KDM5B. promotes tumor proliferation and metastasis	[265]
FOXM1	Upregulation	/	Promotes the stabilization and activation of FOXM1; aggravates the malignant behavior of tumor cells	[266, 267]
p120	Upregulation	/	Promotes tumor cell dissociation and distant metastasis	[268]
β‐Catenin	Upregulation	/	Promotes tumor cell dissociation and distant metastasis	[268]
Cofilin	Upregulation	Ser108	Promotes tumor cell motility and invasion	[269, 270, 271]
MEK2	Upregulation	Thr13	Promotes the proliferation and metastasis of breast cancer cells by stabilizing and activating MEK2	[272]
GATAD2B	Upregulation	/	Inhibits ubiquitination and degradation of GATAD2B; enhances CSC self‐renewal and metastatic drug resistance	[273, 274, 275, 276]
KLF8	Upregulation	/	Promotes CSC phenotypes and chemotherapy resistance	[277, 278]
Abnormal O‐GlcNAcylation is implicated in the pathogenesis of CVDs				
c‐Myc	Upregulation	/	Promotes pathological cardiac remodeling	[306, 307, 308, 309]
Troponin I	Upregulation	/	Promotes pathological cardiac remodeling	[306, 307, 308, 309]
Troponin T	Upregulation	/	Promotes pathological cardiac remodeling	[306, 307, 308, 309]
PKAc	Upregulation	/	Promotes myocardial hypertrophy and dysfunction	[313, 314]
Abnormal O‐GlcNAcylation promotes the pathogenesis of immune‐related diseases				
FOXP3	Downregulation	/	Inhibits Treg cell infiltration and promotes AIH pathology	[319]
NF‐κB	Upregulation	/	Promotes the production of Th17 cells and related inflammatory responses, advancing the MS pathological process	[322, 323, 324]
p65	Upregulation	/	Exacerbates RA progression	[327, 328]
ACC1	Upregulation	/	Enhances the activation of transcription factor RORγt; promotes the proinflammatory effects of Th17 cells; promotes the pathological process of RA	[330, 331]
NFAT	Upregulation	/	Promotes the activation of T cells and B cells	[338, 339, 342]
c‐Myc	Upregulation	/	Promotes T cell activation	[338, 339, 342]
NF‐κB	Upregulation	/	Promote the activation of T cells and B cells	[338, 339, 342]
RelA	Upregulation	Thr305	Promotes the release of inflammatory factors	[342, 350]
c‐Rel	Upregulation	Ser350	Promotes the release of inflammatory factors	[342, 350]
STAT3	Upregulation	Thr717	Inhibits IL‐10 function and promote inflammatory response	[351, 352, 353]
EZH2	Upregulation	Ser75	Promotes NK cell activation and stabilization	[354, 355, 356]
FOXO1	Upregulation	/	Promotes the cytotoxic effect of NK cells	[357, 358]

### 3.2 Abnormal O‐GlcNAcylation Drives Cancer Progression

In recent years, the incidence and mortality rates of cancers have continued to rise, making them a major threat to human health. The development and progression of cancers involve dysregulation of multiple biological processes, including gene mutations, metabolic disorders, and immune evasion, resulting in complex and diverse pathological mechanisms. O‐GlcNAcylation, as an important posttranslational modification of proteins, plays a widespread role in regulating key biological activities such as cellular signal transduction, gene expression, energy metabolism, and immune surveillance. Dysregulated O‐GlcNAcylation is closely associated with the occurrence of various diseases. A growing body of research indicates that O‐GlcNAcylation levels are significantly elevated in various highly prevalent and lethal malignant tumors, including lung cancer, liver cancer, colorectal cancer (CRC), and breast cancer, suggesting its potential critical role in tumorigenesis and progression. However, the molecular mechanisms by which O‐GlcNAcylation promotes the progression of these tumors have not yet been fully elucidated. This section aims to review and discuss recent advances in the regulatory mechanisms of O‐GlcNAcylation in common cancers, and to providing a theoretical basis for related targeted therapeutic strategies.

Lung cancer is one of the most frequently diagnosed malignancies worldwide and a leading cause of cancer‐related mortality [205]. Its development and progression are closely associated with various molecular abnormalities. In recent years, growing evidence has shown that aberrant O‐GlcNAcylation plays a key role in lung cancer progression by influencing various biological processes such as gene expression, signal transduction, and metabolic reprogramming, thereby enhancing tumor proliferation, invasion, metastasis, and therapy resistance [206, 207, 208, 209, 210]. O‐GlcNAcylation significantly enhances oncogenic transcriptional activity by regulating key transcription factors and coactivators. For instance, O‐GlcNAcylation at Ser521 of the nuclear receptor coactivator RBM14 enhances its transcriptional activity, promoting the proliferation and metastasis of lung cancer cells [211, 212, 213]. Similarly, O‐GlcNAcylation at Ser103 of the transcription factor NRF2 by OGT prevents its binding to KEAP1 and subsequent ubiquitin–proteasome degradation, thereby stabilizing and activating NRF2 [214]. This leads to enhanced transcription of downstream antioxidant and prosurvival genes, exacerbating malignant progression and chemotherapy resistance in lung cancer cells [215, 216]. In non‐small cell lung cancer (NSCLC), O‐GlcNAcylation also increases the stability and activity of several transcription‐related proteins, including c‐Myc [217], the nuclear RNA‐binding protein SAM68 [218], and the transcriptional coactivator complex subunit YEATS2 [219], further promoting tumor invasion and metastasis. Beyond transcriptional regulation, O‐GlcNAcylation is directly involved in tumor metabolic reprogramming. Phosphoribosyl pyrophosphate synthetase 1 (PRPS1), a key enzyme in the de novo nucleotide synthesis pathway [220], exhibits enhanced enzymatic activity when O‐GlcNAcylated at Ser83 and Thr166. This promotes nucleotide synthesis, thereby supporting rapid tumor cell proliferation, growth, and drug resistance [221]. Notably, O‐GlcNAcylation also plays a crucial role in maintaining lung cancer stem cell (CSC) properties. The inflammatory cytokine IL‐8, which is highly expressed in lung cancer [222, 223], upregulates glucose transporters GLUT3 and GFAT, facilitating glucose influx into HBP and elevating overall O‐GlcNAcylation levels. This modification helps establish and sustain CSC characteristics, promoting distant metastasis. Inhibition of OGT with OSMI1 significantly reduces CSC populations and suppresses xenograft tumor formation, underscoring the necessity of O‐GlcNAcylation in this process [224]. Furthermore, O‐GlcNAcylation is broadly involved in other malignant behaviors of lung cancer through multiple signaling pathways. For example, the CARM1–USP9X–OGT axis enhances glycolysis and the Warburg effect, promoting tumor growth [225]; O‐GlcNAcylation of SMAD4 activates the TGF‐β signaling pathway to induce epithelial–mesenchymal transition (EMT) [226]; and the IL‐6/STAT3 pathway, regulated by O‐GlcNAcylation, amplifies inflammatory responses, further driving invasion and metastasis [227]. In summary, aberrantly elevated O‐GlcNAcylation drives the initiation and progression of lung cancer through multimolecular and multipathway synergism, making it a promising therapeutic target.

Abnormal O‐GlcNAcylation plays a critical role in the initiation and progression of hepatocellular carcinoma (HCC), one of the most common malignant tumors worldwide [228]. The proportion of HCC cases associated with metabolic dysfunction‐associated steatotic liver disease (MASLD) has been increasing steadily in recent years [229]. A study of 18 MASLD‐HCC patients revealed that 12 exhibited significantly elevated expression of OGT, indicating a strong correlation between aberrant O‐GlcNAcylation and the pathogenesis of MASLD‐HCC [230]. In MASLD‐HCC, highly expressed OGT promotes tumor progression through dual signaling pathways. On one hand, it enhances the expression of p‐JNK and p‐c‐Jun, activates AP‐1, and thereby facilitates JNK/c‐Jun/AP‐1 signal transduction, exacerbating endoplasmic reticulum stress [231, 232]. On the other hand, OGT upregulates the expression of proteins such as p‐IKKa/IKKb, p‐p65, and p‐p50, and enhances the DNA‐binding activity of NF‐κB, promoting palmitic acid metabolism. These changes collectively lead to significantly enhanced growth capacity (*p* < 0.001), clonogenicity (*p* < 0.01), and migration and invasion abilities (*p* < 0.05) in MASLD‐HCC cells [230]. Furthermore, fatty acid synthase (FASN), a key enzyme in fatty acid biosynthesis, also participates in regulating liver cancer progression under elevated O‐GlcNAcylation levels. In HepG2 cells, high O‐GlcNAcylation markedly promotes fatty acid synthesis by activating the PI3K/AKT/mTOR signaling axis, thereby accelerating tumor cell proliferation [233]. Noncoding RNA molecules are also involved in this regulatory network. X‐inactive specific transcript (XIST), a long noncoding RNA highly expressed in liver cancer, promotes O‐GlcNAcylation of the RAF1 oncogene through the XIST/miR‐424‐5p/OGT axis [234, 235]. This modification inhibits ubiquitination‐mediated degradation of RAF1, enhances its stability and activity, and ultimately facilitates liver cancer proliferation and EMT [235]. Additionally, the transcriptional coactivator Yes‐associated protein (YAP), a downstream effector of the Hippo signaling pathway and a known key oncogenic driver, is particularly active under hyperglycemic conditions [236, 237]. Studies confirm that OGT directly modifies the Thr241 residue of the YAP protein, increasing its stability. O‐GlcNAcylated YAP further exacerbates hyperglycemia and hepatic glucose metabolic burden, thereby mediating hyperglycemia‐stimulated hepatocarcinogenesis [238, 239]. In summary, abnormal O‐GlcNAcylation significantly promotes hepatocarcinogenesis, proliferation, invasion, and metastasis by regulating multiple critical biological processes, including gene expression, signal transduction, fatty acid synthesis, and glucose metabolism. It represents a core molecular mechanism in HCC progression.

Abnormal O‐GlcNAcylation plays a critical role in the initiation and progression of CRC, the fourth deadliest cancer worldwide, causing approximately 90,000 deaths annually [240]. The malignant progression of CRC is closely associated with dysregulation of various molecular mechanisms, among which aberrant elevation of O‐GlcNAcylation has become a major research focus. Studies have shown that in CRC cell lines HT29 and HCT116, the overall O‐GlcNAcylation level, OGT, and GFAT—the rate‐limiting enzyme of HBP—are significantly higher than in normal colon cells [241]. Furthermore, dozens of proteins in CRC tissues exhibit abnormally high O‐GlcNAcylation, which suggests a strong correlation between upregulated O‐GlcNAcylation and CRC pathogenesis [242]. In nucleic acid metabolism, thymidylate synthase (TS), a key enzyme in de novo dTMP synthesis essential for DNA synthesis and damage repair, is O‐GlcNAcylated at Thr251 and Thr306 by highly expressed OGT in CRC. This modification inhibits TS proteasomal degradation, enhances its stability, and ultimately promotes tumor cell proliferation [243]. At the gene expression level, the transcription factor YY1, overexpressed in various cancers including CRC [244], is stabilized through O‐GlcNAcylation at Thr236. Modified YY1 activates the expression of oncogenes SLC22A15 and AANAT, facilitating tumorigenesis [245]. Additionally, OGT modifies the Ser196‐198 sites of the m6A reader protein YTHDF1, strengthening its interaction with exportin protein Crm1, promoting its cytoplasmic localization and translation of the downstream oncoprotein c‐Myc, thereby driving cell proliferation [246, 247, 248]. O‐GlcNAcylation also directly upregulates the expression of oncogenes such as casein kinase 2 alpha 1 (CSNK2A1) [249]. These findings demonstrate that O‐GlcNAcylation promotes CRC progression by regulating transcription, translation, and oncoprotein stability. Metabolic reprogramming is a hallmark of cancer. The Warburg effect—enhanced aerobic glycolysis—commonly observed in CRC is also regulated by O‐GlcNAcylation [250, 251]: OGT mediates glycosylation of c‐Myc at Ser415, enhancing its stability and activity, which upregulates PDK2, suppresses mitochondrial pyruvate metabolism and ROS generation, ultimately reinforcing glycolysis, inhibiting the tricarboxylic acid cycle, and promoting tumor cell proliferation [252]. In lipid metabolism, FASN is highly expressed in CRC [253] and positively regulates the expression of OGT and GFPT1, further elevating O‐GlcNAcylation levels and forming a positive feedback loop that accelerates tumor growth and metastasis [254]. Cell–cell and cell–matrix interactions are also modulated by O‐GlcNAcylation. Integrin α5 (ITGA5) shows enhanced stability after O‐GlcNAcylation, promoting cell adhesion, invasion, and EMT [255, 256, 257, 258, 259]. Moreover, the activity and stability of EZH2 are increased due to elevated O‐GlcNAcylation, leading to altered expression of E‐cadherin and β‐catenin and further facilitating tumor metastasis [260]. In the tumor immune microenvironment (TME), the phenotypic polarization of tumor‐associated macrophages (TAMs) is influenced by O‐GlcNAcylation. In colorectal adenocarcinoma (COAD), OGT collaborates with the deubiquitinating enzyme USP18 to inhibit STAT2 degradation, promoting M2 polarization of TAMs and supporting tumor immune evasion and growth [261]. Finally, studies have revealed that SET domain‐containing protein 5 in CRC upregulates the expression of CSC markers such as ESRRB, CD133, and KLF4 by facilitating OGT‐mediated O‐GlcNAcylation of RNAP II, endowing tumor cells with stem‐like properties—a process dependent on O‐GlcNAcylation [262]. In summary, abnormal O‐GlcNAcylation comprehensively drives CRC progression by regulating gene expression, nucleic acid and metabolic reprogramming, fatty acid synthesis, cellular interactions, immune microenvironment remodeling, and cancer stemness, underscoring its pivotal role as a core molecular mechanism in CRC.

Breast cancer, a global health concern, ranks among the top malignancies in women in terms of both incidence and mortality, posing a serious threat to female lives. Recent studies have revealed that aberrant O‐GlcNAcylation plays a key role in promoting the development and progression of breast cancer. In breast cancer, highly expressed TGF‐β1 enhances the stability of GFAT (the rate‐limiting enzyme of the HBP) and activates the mTOR/MYC signaling cascade. This cascade ultimately upregulates OGT expression and elevates global O‐GlcNAcylation levels [263, 264]. Substantial evidence indicates that O‐GlcNAcylation is abnormally elevated in breast cancer and significantly promotes disease progression. On one hand, O‐GlcNAcylation influences gene expression by regulating key transcription factors and chromatin‐remodeling proteins. For example, the Thr556 residue of the chromatin remodeler MORC2 can be O‐GlcNAcylated, and modified MORC2 promotes the expression of TGF‐β1 downstream target genes CTGF and SNAIL, both of which are critical for breast cancer invasion and metastasis [263]. Inhibiting O‐GlcNAcylation markedly reduces the invasive ability of breast cancer cells, indicating that this modification drives tumor progression through MORC2‐mediated transcriptional mechanisms [263]. In triple‐negative breast cancer, O‐GlcNAcylation at Ser40 of histone H2A inhibits the histone demethylase KDM5B, thereby promoting tumor proliferation and metastasis [265]. Additionally, the stability of the oncogenic transcription factor FOXM1 is regulated by O‐GlcNAcylation: under normal conditions, FOXM1 undergoes SIRT1‐mediated ubiquitination and degradation, whereas high levels of O‐GlcNAcylation in breast cancer inhibit the AMPK–SIRT1 axis, enhancing FOXM1 stability and exacerbating malignant behavior [266, 267]. On the other hand, O‐GlcNAcylation promotes tumor progression by affecting cell adhesion, motility, and signal transduction. For instance, OGT overexpression modifies p120 and β‐catenin, leading to reduced E‐cadherin on the cell surface, weakened intercellular adhesion, and facilitated tumor cell detachment and distant dissemination [268]. Meanwhile, O‐GlcNAcylation at Ser108 of the motility‐related protein cofilin enhances its stability, promoting cell movement and invasion [269, 270, 271]. MEK2, an upstream kinase in the ERK signaling pathway, undergoes O‐GlcNAcylation at Thr13, which promotes Thr394 autophosphorylation, enhancing MEK2 stability and activation, thereby driving breast cancer cell proliferation. Mutation at this site significantly reduces tumor proliferation and metastasis [272]. Furthermore, O‐GlcNAcylation promotes drug resistance and metastasis by maintaining CSC properties. GATAD2B, a component of the NuRD complex and a substrate of OGT, is stabilized through O‐GlcNAcylation, which inhibits its ubiquitination and degradation, thereby enhancing CSC self‐renewal and metastatic drug resistance [273, 274, 275, 276]. Similarly, KLF8, a key regulator of mammosphere formation in breast cancer, is also modified by O‐GlcNAcylation, further promoting CSC phenotypes and chemotherapy resistance [277, 278]. In summary, aberrantly elevated O‐GlcNAcylation in breast cancer drives disease progression through multiple mechanisms, including enhancing oncogene expression, disrupting cell adhesion, activating motility‐related proteins, and maintaining stem cell properties, collectively promoting tumor proliferation, invasion, metastasis, and drug resistance. Notably, upregulated O‐GlcNAcylation significantly facilitates EMT and distant metastasis in breast cancer.

In summary, aberrant O‐GlcNAcylation not only significantly promotes the progression of highly prevalent and lethal malignancies such as lung cancer, liver cancer, CRC, and breast cancer, but also plays a critical role in the development and progression of various other malignant solid tumors, including thyroid [279, 280, 281], gastric [282, 283, 284], esophageal [285], cervical [286, 287, 288, 289, 290], ovarian [291, 292, 293, 294, 295], and renal [296, 297] cancers. Thus, abnormal O‐GlcNAcylation can disrupt protein functions, thereby affecting core biological processes such as gene expression, signal transduction, metabolic regulation, and immune modulation, broadly driving the proliferation, invasion, and metastasis of epithelial‐derived malignancies (Table 2).

### 3.3 Abnormal O‐GlcNAcylation is Implicated in the Pathogenesis of CVDs

CVDs are the leading cause of death and disability worldwide [298, 299, 300]. In recent years, growing evidence has shown that the occurrence and progression of CVDs are closely associated with abnormal levels of O‐GlcNAcylation [301, 302, 303, 304].

Elevated O‐GlcNAcylation plays a key role in the development and progression of cardiac hypertrophy and heart failure (HF) [305]. Cardiac hypertrophy is a typical pathological response of the heart to chronic overload, commonly triggered by conditions such as aortic stenosis (AS), aortic coarctation, and hypertension. These conditions can ultimately lead to severe clinical outcomes including HF and myocardial infarction (MI). Multiple studies have confirmed that O‐GlcNAcylation levels are significantly increased across various heart disease models. In patients with AS, both OGT protein and mRNA expression levels were markedly elevated, with an overall increase of 64.7 ± 7.6% in protein O‐GlcNAcylation compared with the control group. In rat models of aortic coarctation‐induced cardiac hypertrophy and HF, O‐GlcNAcylation levels increased by 80.6  ± 13.1% and 58.2 ±10.0%, respectively. Additionally, hypertensive rat models showed a 47.2 ± 6.5% rise in O‐GlcNAcylation [305]. These data consistently indicate a significant positive correlation between elevated O‐GlcNAcylation and pathological conditions such as AS, aortic coarctation, and hypertension. Functional studies further reveal the pathogenic role of O‐GlcNAcylation in cardiac pathology. Even in the absence of pathological stress, transgenic overexpression of OGT in mouse hearts was sufficient to induce ventricular arrhythmias and premature death. Conversely, reducing O‐GlcNAcylation by overexpressing OGA alleviated cardiac injury and maintained heart function, demonstrating that O‐GlcNAcylation itself has direct disease‐promoting effects [303].

At the molecular level, elevated O‐GlcNAcylation promotes pathological cardiac remodeling through multiple pathways. For instance, excessive O‐GlcNAc modification of key proteins such as c‐Myc, troponin I, and troponin T exacerbates the process of cardiac remodeling [306, 307, 308, 309]. Furthermore, in HF and cardiomyopathy, widespread protein modifications mediated by OGT suppress the activity of genes related to mitochondrial metabolism, suggesting a potential role in promoting cardiac decompensation by impairing mitochondrial function [303]. Additionally, increased O‐GlcNAcylation activates the mTOR signaling pathway, which is known to be closely associated with cardiomyocyte growth and hypertrophy [310, 311], thereby further aggravating pathological cardiac hypertrophy and HF [312]. Similarly, this modification can induce a hypertrophic phenotype by activating the protein kinase A (PKA) pathway. A study by Chen et al. demonstrated that OGT directly modifies the PKA catalytic subunit PKAc via O‐GlcNAcylation. The modified PKAc promotes the synthesis of atrial natriuretic peptide and β‐myosin heavy chain, thereby driving cardiac hypertrophy and dysfunction [313, 314]. Abnormally elevated O‐GlcNAc levels have also been detected in animal models of MI. More importantly, in a postinfarction HF model, further increasing O‐GlcNAcylation levels with the OGA inhibitor PUGNAc led to a significant decline in cardiac contractile function, reaffirming the causal relationship between elevated modification levels and worsening cardiac function [305]. Thus, abnormally elevated O‐GlcNAcylation significantly promotes the development of cardiac hypertrophy, HF, and other cardiovascular pathologies by affecting multiple critical signaling pathways and cellular processes, making it an important pathogenic factor.

The aforementioned study indicates that abnormal O‐GlcNAcylation levels are closely associated with the development and progression of CVDs, but its role is bidirectional. On one hand, substantial evidence suggests that high levels of O‐GlcNAcylation significantly promote CVDs; on the other hand, multiple studies have also pointed out that its low expression similarly accelerates CVDs progression. Specifically, an abnormal decrease in O‐GlcNAcylation is associated with an increased risk of major adverse cardiovascular events (MACEs; including cardiovascular death, nonfatal acute MI, and nonfatal ischemic stroke). A 2025 retrospective study divided 1947 CVD patients into a high‐expression group (974 cases) and a low‐expression group (973 cases) based on their average serum OGT levels. After long‐term follow‐up, the MACEs incidence in the high‐expression group was 7.6% (74 cases), significantly lower than the 10.4% (100 cases) in the low‐expression group (*p* = 0.032), suggesting that low O‐GlcNAcylation expression may increase the risk of MACEs [315]. Moreover, consistent trends were observed in animal models. A study using a tamoxifen‐induced MI model compared the outcomes between OGT‐knockout mice and wild‐type C57BL/6 mice. The results showed that 4 weeks after MI, the survival rate in the OGT‐knockout group was 64%, significantly lower than the 80% in the wild‐type group, further indicating that the loss of O‐GlcNAcylation may exacerbate the progression of HF [316]. These findings demonstrate that both excessive elevation and abnormal reduction of O‐GlcNAcylation may promote the development of CVDs through different mechanisms.

In summary, experimental observations and molecular mechanism studies indicate that the level of O‐GlcNAcylation significantly influences the occurrence and progression of CVDs. High expression of O‐GlcNAcylation markedly promotes pathological processes such as AS, arterial constriction, and hypertension, thereby exacerbating HF, myocardial hypertrophy, and ischemic myocardial injury (Table 2). Conversely, its low expression also contributes to the occurrence of MACEs and accelerates the deterioration of HF. These findings suggest that maintaining the homeostasis of O‐GlcNAcylation is crucial for the structural and functional integrity of the cardiovascular system. Abnormal expression levels—whether elevated or reduced—may significantly drive the development and progression of CVDs.

### 3.4 Abnormal O‐GlcNAcylation Promotes the Pathogenesis of Immune‐Related Diseases

O‐GlcNAcylation is a crucial posttranslational modification essential for normal immune system function. It contributes to immune homeostasis by precisely regulating the functions of various immune cells, the activity of immune molecules, and related biological processes. However, aberrant O‐GlcNAcylation can disrupt immune homeostasis and trigger the onset or exacerbation of various immune‐related diseases. For instance, in autoimmune diseases and organ transplant rejection, aberrant O‐GlcNAcylation promotes pathogenesis by influencing immune cell activation and infiltration and modulating inflammatory factor secretion. This section will systematically elaborate on the specific mechanisms by which abnormal O‐GlcNAcylation contributes to the pathogenesis and progression of these diseases.

Autoimmune hepatitis (AIH) is an immune‐mediated inflammatory disease of the liver parenchyma, characterized pathologically by CD4⁺ and CD8⁺ T lymphocyte infiltration leading to hepatocyte injury [317, 318]. As a key organ in maintaining immune homeostasis, the liver promotes immune tolerance to hepatocyte autoantigens by facilitating the infiltration of regulatory T cells (Tregs), thereby suppressing autoimmune responses. In AIH patients, the number of Tregs is significantly reduced, and their immunoregulatory function is critically dependent on the expression of the transcription factor FOXP3. The expression of FOXP3 is regulated by multiple mechanisms, among which O‐GlcNAcylation serves as an important regulatory pathway. Comparative studies using Eogt^−^⁄^−^ and Eogt⁺⁄⁺ mouse models revealed that Eogt^−^⁄^−^ mice lacking O‐GlcNAcylation modification exhibited more severe AIH pathological phenotypes, suggesting a protective role of this modification in suppressing AIH pathogenesis. Further molecular mechanism studies indicated that O‐GlcNAcylation promotes FOXP3 expression by activating the Notch signaling pathway, thereby enhancing Tregs differentiation and liver infiltration. However, under AIH conditions, abnormally reduced O‐GlcNAcylation levels lead to decreased Notch signaling activity, restricted FOXP3 expression, impaired Treg function, and insufficient infiltration, ultimately exacerbating autoimmune responses and promoting AIH progression [319]. Thus, abnormally reduced O‐GlcNAcylation contributes to the development and progression of AIH by impairing Treg function and infiltration.

Multiple Sclerosis (MS) is an inflammatory autoimmune disorder characterized by chronic demyelination and neurodegenerative changes in the central nervous system. Its core pathological mechanism involves the differentiation of CD4⁺ T cells into the Th17 subset and the excessive production of IL‐17, which mediates autoimmune inflammatory responses [320, 321]. In this process, microRNA‐15b, a member of the microRNA family, plays a critical regulatory role. This molecule is aberrantly expressed in various autoimmune diseases, neurodegenerative diseases, and cancers. Studies have shown that miR‐15b expression is significantly downregulated in CD4⁺ T cells of MS patients, and its level is negatively correlated with Th17 cell differentiation. Mechanistic studies further demonstrate that reduced miR‐15b expression enhances Th17 cell differentiation and infiltration, thereby driving MS progression. At the molecular level, this regulation depends on O‐GlcNAcylation modification of the nuclear transcription factor NF‐κB by OGT. When miR‐15b expression decreases, this modification process is enhanced, boosting NF‐κB transcriptional activity and upregulating its downstream target gene retinoic acid‐related orphan receptor γt (RORγt). As a key transcription factor for Th17 cell differentiation, increased RORγt expression further promotes Th17 cell generation and related inflammatory responses, ultimately exacerbating MS pathogenesis [322, 323, 324]. Therefore, the aberrantly elevated O‐GlcNAcylation of NF‐κB significantly promotes the pathogenesis of MS by modulating Th17 cell function.

Rheumatoid arthritis (RA) is an autoimmune disease characterized by synovitis and bone damage [325]. Its pathological mechanism involves abnormal activation of inflammatory factors and immune cell infiltration, and while the exact process remains incompletely understood, studies indicate a close association with O‐GlcNAcylation [326]. During the onset of RA, TNFα, a key regulator of inflammation, promotes O‐GlcNAcylation events during osteoclastogenesis. Research by Kim et al. demonstrated that TNFα enhances O‐GlcNAcylation of the transcription factor p65. This modification increases p65's DNA‐binding capacity and transcriptional activity, thereby exacerbating RA progression [327, 328]. Additionally, IL‐1β plays a significant role in sustaining chronic inflammation in RA. It induces O‐GlcNAcylation of downstream signaling molecules, further driving disease development [329]. Similar to MS, RA joint tissues exhibit extensive infiltration of Th17 cells differentiated from CD4+ T cells. Studies show that O‐GlcNAcylation of acetyl‐CoA carboxylase 1 (ACC1) in RA enhances the activation of transcription factor RORγt. This, in turn, promotes the production of the highly proinflammatory cytokine IL‐17A by Th17 cells, ultimately intensifying the inflammatory response [330, 331]. Thus, O‐GlcNAcylation significantly contributes to RA progression by participating in inflammatory factor regulatory networks and promoting immune cell infiltration and cytokine release, making it a potential target for therapeutic intervention.

Organ transplantation is a critical treatment for end‐stage organ failure, yet it is often complicated by various postoperative issues, with immune rejection representing the most significant and potentially fatal complication. Similar to autoimmune diseases, this process is primarily driven by immune cell‐mediated inflammatory infiltration and tissue damage. Notably, the mTOR signaling pathway is frequently hyperactivated in transplant recipients and regulates multiple cellular processes, including metabolism and O‐GlcNAcylation [332]. As an important posttranslational modification, O‐GlcNAcylation is widely involved in the differentiation, maturation, and functional regulation of immune cells such as T cells, B cells, macrophages, and NK cells, playing a significant role in transplant immune rejection. First, O‐GlcNAcylation exacerbates cellular immune responses by promoting T cell maturation and activation while inhibiting apoptosis. Studies show that during T cell receptor (TCR) β‐chain rearrangement, knockdown of OGT leads to a significant reduction in CD4⁺ CD8⁺ T cells, while OGT overexpression promotes their generation, indicating that O‐GlcNAcylation levels directly affect TCR rearrangement efficiency and T cell development [333, 334, 335]. During activation, OGT expression markedly increases in T cells [336, 337]; siRNA‐mediated OGT inhibition significantly reduces the synthesis and release of the key activator IL‐2 [338, 339, 340, 341]. O‐GlcNAcylation also modulates the activity of critical transcription factors involved in T cell activation, such as NFAT, c‐Myc, and NF‐κB, whose activation is essential for IL‐2 transcription [338, 339, 342]. Furthermore, reducing O‐GlcNAcylation levels upregulates PD‐1 expression and promotes CD8⁺ T cell apoptosis, whereas overexpression exerts an antiapoptotic effect [343, 344]. These findings collectively demonstrate that O‐GlcNAcylation enhances T cell‐mediated immune responses in multiple ways. Second, O‐GlcNAcylation significantly promotes B cell survival, activation, and antibody production. B cell survival depends on the BAFF/BAFFR signaling pathway. OGT deficiency reduces the expression of key molecules—p50, p65, RelB, and p52—leading to B cell apoptosis. [345, 346, 347]. Under low OGT conditions, the proportions of splenic follicular B cells and mature B cells in mice decrease, and the activity of germinal center B cells, memory B cells, and plasma cells is suppressed, indicating that O‐GlcNAcylation is essential for maintaining B cell homeostasis and antibody responses [348]. Conversely, OGT overexpression enhances O‐GlcNAcylation of NF‐κB p65 and NFATc1, further elevating B cell activation [349]. Thus, O‐GlcNAcylation significantly promotes antibody‐mediated rejection by strengthening humoral immune responses. Finally, O‐GlcNAcylation broadly regulates the functions of innate immune cells such as macrophages and NK cells, exacerbating inflammatory responses. In macrophages, OGT‐mediated O‐GlcNAcylation of NF‐κB subunits RelA and c‐Rel at Thr305 and Ser350 enhance their transcriptional activity and stability, promoting the release of inflammatory factors such as NO and ILs [342, 350]. Meanwhile, O‐GlcNAcylation of STAT3 at Thr717 can suppress the production or signaling of the anti‐inflammatory cytokine IL‐10, further amplifying proinflammatory responses [351, 352, 353]. In NK cells, O‐GlcNAcylation at the Ser75 site of histone methyltransferase EZH2 promotes cell activation and homeostasis [354, 355, 356]. Additionally, O‐GlcNAcylation inactivates the transcription factor FOXO1 by promoting its de‐phosphorylation, thereby relieving its suppression of NK cell function and enhancing cytotoxicity [357, 358]. Clearly, O‐GlcNAcylation plays a central role in immune rejection after organ transplantation. By promoting the activation, survival, and function of T and B cells, as well as enhancing the inflammatory effects of macrophages and NK cells, it drives cellular, humoral, and innate immune responses in multiple dimensions, ultimately leading to rejection. Targeting the O‐GlcNAcylation signaling pathway may offer new strategies for antirejection therapy.

In summary, the stability of O‐GlcNAcylation levels is crucial for maintaining immune system homeostasis. Under physiological conditions, moderate O‐GlcNAcylation supports the normal functioning of the immune regulatory network. However, abnormal O‐GlcNAcylation can significantly disrupt immune balance, leading to functional disorders and abnormal infiltration of various immune cells—such as T cells, B cells, NK cells, and macrophages—thereby promoting the development and progression of multiple immune‐related diseases (Table 2). These include AIH, MS, RA, and organ transplant rejection.

## 4 Prospects of Targeted O‐GlcNAcylation Therapy

O‐GlcNAcylation plays a critical role in the development and progression of various diseases, including neurodegenerative diseases, cancers, CVDs, and immune‐related disorders. Given its significant contribution to pathological processes, targeting O‐GlcNAcylation demonstrates broad clinical application prospects. However, systematic exploration and comprehensive summaries focusing on this target remain relatively limited. This section aims to discuss the feasibility of O‐GlcNAcylation as a therapeutic target, along with current challenges and future research directions. The goal is to provide new perspectives for optimizing clinical treatment strategies and advancing drug development for related diseases.

### 4.1 Feasibility of O‐GlcNAcylation as a Therapeutic Target

Abnormal O‐GlcNAcylation is widely involved in the occurrence and progression of various diseases, playing a significant role in promoting pathological processes. Its expression and functional impact vary considerably across different diseases: in cancers and CVDs, aberrant elevation of O‐GlcNAcylation markedly accelerates disease progression, whereas in neurodegenerative and immune‐related disorders, either increases or decreases in its levels can exacerbate pathogenesis. Overall, O‐GlcNAcylation serves as a key regulatory mechanism in multiple diseases, and its dysregulation is an important driver of disease development. Therefore, targeting O‐GlcNAcylation offers a promising strategy to effectively halt disease progression and has important implications for optimizing clinical treatment approaches. In eukaryotes, O‐GlcNAcylation is catalyzed by two highly conserved enzymes: OGT and OGA. The absence of isoenzymes means that interventions targeting OGT and OGA avoid issues such as functional redundancy, off‐target effects, or reduced efficacy, thereby ensuring high specificity. Moreover, the sole substrate for this modification, UDP‐GlcNAc, is exclusively supplied by HBP. Targeting HBP does not trigger metabolic compensation or alternative pathways. Thus, for diseases highly dependent on O‐GlcNAcylation, interventions aimed at OGT, OGA, or HBP not only represent promising therapeutic strategies but also demonstrate broad prospects for clinical translation.

OGT is a glycosyltransferase that covalently modifies target proteins by catalyzing O‐GlcNAcylation, playing a role in regulating various cellular processes. To date, several classes of OGT inhibitors have been developed, including UDP‐GlcNAc analogs (such as Alloxan [359, 360, 361, 362, 363, 364], UDP‐5S‐GlcNAc [365, 366, 367], BADGP [368, 369, 370, 371, 372], and Ac‐5SGlcNAc [373, 374, 375]), high‐throughput screening‐derived inhibitors (such as ST045849 [376, 377, 378, 379, 380], BZX [381, 382], and the OSMI series [383, 384, 385, 386, 387, 388, 389]), bisubstrate inhibitors (such as goblin1‐2 [390, 391]), and benzoxazolinones [392] (Table 3). However, these inhibitors generally suffer from limitations such as low specificity, high cytotoxicity, poor cell permeability, or significant off‐target effects. Among them, the small molecule OSMI is widely used in disease mechanism research and preclinical models due to its relatively stable physicochemical properties, low side effects, and good cellular permeability. Particularly in malignant tumor research, OSMI demonstrates multifaceted inhibitory effects. For example, in prostate cancer, OSMI effectively inhibits OGT‐mediated super‐enhancer‐dependent gene expression and the function of KIF1A protein, which drives neuroendocrine differentiation, thereby significantly suppressing tumor cell proliferation [393, 394]. In pancreatic ductal adenocarcinoma, OGT promotes tumor recurrence by modifying and stabilizing the transcription factor SOX2, while OSMI treatment markedly reduces the O‐GlcNAcylation level and stability of SOX2, thus decreasing recurrence [395]. In NSCLC, OSMI enhances the sensitivity of STK11/KRAS/LKB1‐mutant tumors to cisplatin by interfering with OGT's regulation of the HBP [396]. Furthermore, multiple studies have confirmed that OSMI, either alone or in combination with chemotherapeutic agents, significantly induces apoptosis in prostate cancer cell line PC‐3 [397], CRC cell lines HCT116 and LS174T [398, 399], liver cancer cell lines HCC and HepG2 [400, 401, 402, 403], and bladder cancer cell line YTS‐1 [404]—an effect also validated in vitro. Beyond malignant tumors, OSMI shows potential therapeutic value in CVDs. High OGT activity catalyzes O‐GlcNAcylation at Ser280 of CaMKII, triggering massive mitochondrial ROS release and leading to ventricular cardiomyocyte injury [405]. OSMI intervention effectively inhibits this modification process and alleviates ROS‐mediated myocardial damage. Thus, OGT demonstrates significant potential as a therapeutic target in both cancers and CVDs, and its inhibitor OSMI exhibits broad biological activity and promising application prospects in basic and preclinical research. Although more data are needed to confirm the effectiveness of OGT as a therapeutic target in other diseases with abnormally high O‐GlcNAcylation, existing evidence provides a strong basis for its further development.

**TABLE 3:** Reagents for regulating protein O‐GlcNAcylation levels.

Reagent	Type	Advantage	Disadvantage	References
OGT inhibitors (downregulation of O‐GlcNAcylation)				
Alloxan (2,4,5,6‐tetraoxypyrimidine)	Substrate analogues	Early tools for exploring the biological functions of O‐GlcNAc	Lacks of specificity; off‐target effects, promotes ROS generation; cytotoxicity; leading to apoptosis and necrosis of pancreatic β cells	[359, 360, 361, 362, 363, 364]
UDP‐5S‐GlcNAc	Substrate analogues	Exhibits extremely high specificity	Utilized by OGT at a much lower rate compared with UDP‐GlcNAc; reduces the intracellular pool of UDP‐GlcNAc by hijacking HBP; affects other glycosyltransferases by either direct or indirect inhibition	[365, 366, 367]
BADGP	Substrate analogues	Highly cell permeable	Inhibitory efficacy is low	[368, 369, 370, 371, 372]
Ac‐5SGlcNAc	Substrate analogues	High specificity; highly cell permeable	There has been no evaluation of its efficiency and safety in vivo.	[373, 374, 375]
ST045849 (compound 4)	HTS‐derived inhibitors	Can penetrate cells and possess cellular vitality	Relatively low inhibitory potency and impact on protein stability	[376, 377, 378, 379, 380]
BZX (compound 5)	HTS‐derived inhibitors	Inhibitory efficacy is good and cell viability	Cross‐links the active site of OGT (Lys842 and Cys917) through a double‐displacement mechanism, with potential toxic and off‐target effects	[381, 382]
OSMI series	HTS‐derived inhibitors	High specificity; highly cell permeable	Inhibitory efficacy is low; off‐target effect; incomplete specificity; leads to unreliable reversible inactivation of OGT; affects cell viability	[383, 384, 385, 386, 387, 388, 389]
Goblin1‐2	Bisubstrate inhibitors	Highly specific and glucose concentration‐dependent inhibition	Lacks of cell permeability	^[^ ^390^, ^391^ ^]^
OGA inhibitors (upregulation of O‐GlcNAcylation)				
PUGNAc	OGA inhibitors	Highly cell permeable	Lacks of selectivity and specificity; relatively low inhibitory potency; cytotoxic	[406, 407]
Thiamet‐G	OGA inhibitors	High selectivity and efficiency; highly cell permeable	Nonspecific global impact; cytotoxic; inducible insulin resistance	[408, 409]
NButGT	OGA inhibitors	Highly efficient and little impact on cell viability	Off‐target effects; low blood–brain barrier penetration efficiency; synaptic toxicity	[410, 411, 412]
NAG‐thiazoline	OGA inhibitors	Highly cell permeable; high efficiency and specificity	Short half‐life, unstable structure, easily metabolized and eliminated; lack of selectivity in vivo; easy to produce off‐target effects; cytotoxic	[413]
GlcNAcstatin	OGA inhibitors	Highly efficient	Lacks of cell permeability; metabolized or eliminated more rapidly in the body; off‐target effects	[414, 415]
HBP inhibitors (downregulation of O‐GlcNAcylation)				
Azaserine (O‐diazoacetyl‐l‐serine)	GFAT inhibitors	Highly efficient	Severe systemic toxicity, carcinogenicity, and low selectivity to GFAT	[420]
DON (6‐diazo‐5‐oxo‐l‐norleucine)	GFAT inhibitors	Highly efficient; antitumor proliferation	Has low selectivity for GFAT; causes systemic toxicity	[421, 422, 423, 424, 425]

OGA is a hydrolase that specifically cleaves the covalent bond between O‐GlcNAc and proteins, thereby removing O‐GlcNAc modifications from target proteins, a process known as de‐O‐GlcNAcylation. Reduced levels of O‐GlcNAcylation have been observed in various diseases, including neurodegenerative diseases such as AD, PD, HD, and ALS, as well as certain immune‐related diseases. Therefore, inhibiting OGA to restore normal protein glycosylation levels is considered a potential therapeutic strategy. Several OGA inhibitors have been developed, including PUGNAc [406, 407], Thiamet‐G [408, 409], NButGT [410, 411, 412], NAG‐thiazoline [413], and GlcNAcstatin [414, 415] (Table 3). As the earliest and most widely used inhibitor, PUGNAc has demonstrated protective effects in multiple neurodegenerative disease models: in AD models, it increases O‐GlcNAcylation of Tau protein and reduces its hyperphosphorylation, thereby slowing the decline in learning and memory functions [416]; in PD and ALS models, PUGNAc enhances neuronal activity and delays disease progression [417]. Although PUGNAc shows certain therapeutic effects, its poor substrate selectivity and structural limitations hinder its clinical application [418]. To overcome these drawbacks, researchers developed GlcNAcstatin based on the structure of PUGNAc, which exhibits high selectivity for human OGA (hOGA) [419]. Currently, research on GlcNAcstatin, NButGT, Thiamet‐G, and other inhibitors in diseases associated with O‐GlcNAcylation deficiency remains relatively limited. Nevertheless, the neuroprotective effects demonstrated by PUGNAc fully indicate that targeting OGA for drug intervention is both feasible and worthy of further exploration.

The HBP is the sole metabolic pathway in cells for UDP‐GlcNAc, the substrate required for O‐GlcNAcylation. Inhibiting HBP effectively reduces UDP‐GlcNAc production, thereby decreasing overall protein O‐GlcNAcylation levels. Thus, targeting HBP, particularly its key rate‐limiting enzyme GFAT, represents a promising strategy for correcting abnormal O‐GlcNAcylation. Currently known GFAT inhibitors primarily include glutamine analogs, such as O‐diazoacetyl‐l‐serine (azaserine) [420] and 6‐diazo‐5‐oxo‐l‐norleucine (DON) [421, 422, 423, 424, 425] (Table 3). These compounds not only significantly inhibit tumor cell proliferation and other malignant phenotypes but also demonstrate therapeutic potential in CVDs [426]. Studies have shown that inhibiting GFAT activity with DON or azaserine effectively alleviates CVDs and improves pathological conditions [426]. Notably, a synergistic effect exists between inhibiting HBP and directly inhibiting OGT. Simultaneously targeting both pathways can further enhance the regulation of O‐GlcNAcylation. Therefore, targeting HBP not only helps improve the efficacy of OGT inhibitors but also practically validates the feasibility of targeting HBP in the treatment of diseases associated with elevated O‐GlcNAcylation levels.

In summary, aberrant O‐GlcNAcylation is a significant driver of disease progression. This modification is uniquely dependent on the HBP for substrate supply, with its addition and removal catalyzed exclusively by OGT and OGA, respectively. Due to this well‐defined and streamlined mechanism, O‐GlcNAcylation represents a promising therapeutic target. Indeed, interventions targeting this process in various disease models have successfully attenuated pathological progression and shown promising therapeutic efficacy. Therefore, developing treatment strategies focused on modulating O‐GlcNAcylation is highly feasible.

### 4.2 Current Challenges and Future Directions

O‐GlcNAcylation, as a crucial posttranslational modification of proteins, is extensively involved in various vital biological processes such as cellular signal transduction, transcriptional regulation, and metabolic homeostasis. Its dysregulation is closely associated with the development and progression of multiple diseases. Therefore, targeting the O‐GlcNAc cycling pathway—including inhibiting OGT, OGA, or HBP—has emerged as an important therapeutic strategy. However, this approach still faces several key challenges in translational applications. The primary issue lies in the complex relationship between the biological characteristics of O‐GlcNAcylation and disease pathology. This modification is widespread throughout the body and plays a critical role in maintaining homeostasis, whereas related diseases such as malignant tumors, neurodegenerative diseases, and CVDs often exhibit tissue‐specific manifestations. Thus, an ideal therapeutic strategy must precisely regulate abnormal O‐GlcNAcylation levels at the lesion site while preserving systemic physiological functions, thereby avoiding side effects caused by broad inhibition. Achieving such spatial selectivity remains a major focus and challenge in current research. Second, existing drugs targeting O‐GlcNAcylation still exhibit significant limitations in potency and specificity. Numerous inhibitors developed to date—such as those targeting OGT, OGA, or GFAT—commonly suffer from issues like low target selectivity, poor cell permeability, high cytotoxicity, and off‐target effects. For instance, the classic OGT inhibitor Alloxan not only has limited efficacy but also induces ROS generation leading to cell death and may simultaneously affect OGA activity. Benzoxazolinone‐based molecules can cause irreversible OGT inactivation, which lacks controllability, while BADGP may compromise antipathogen defense by interfering with host immune responses [427, 428]. These limitations underscore the urgent need to develop novel inhibitors with high selectivity, stability, and low toxicity. Although targeting O‐GlcNAcylation still faces numerous obstacles, addressing two key issues—tissue‐specific regulation and drug efficacy/safety—could significantly advance its clinical translation. Future research should focus on designing precise intervention strategies for localized pathological sites and optimizing compound structures to enhance selectivity and biocompatibility, thereby offering new avenues for treating related diseases.

Despite its promise as a therapeutic strategy for various diseases, targeting O‐GlcNAcylation faces two major challenges: the inadequate efficacy of current inhibitors and their lack of spatial specificity. Overcoming these limitations will be a central focus of future research.

To tackle the issue of insufficient inhibitors performance, there are currently two main optimization pathways. The first involves improving performance through drug iteration. This entails systematically optimizing the structure, efficacy, and side effects of previous‐generation drugs to develop next‐generation drugs with stronger inhibitory effects, higher selectivity, and lower toxicity. For example, the OGA inhibitor GlcNAcstatin was optimized based on the structure of PUGNAc, demonstrating superior inhibitory efficiency and specificity for OGA [429, 430]. Similarly, starting from OSMI1, researchers have successively developed more specific follow‐up versions such as OSMI2, OSMI3, and OSMI4. The second pathway involves exploring new types of chemical drugs or biological agents. Currently, interventions for diseases related to abnormal O‐GlcNAcylation levels primarily rely on inhibitors: diseases with high O‐GlcNAcylation often target OGT or GFAT (a rate‐limiting enzyme in the HBP pathway), while those with low levels often inhibit OGA. However, research on corresponding agonists has lagged. In fact, developing agonists for OGT, OGA, or GFAT holds significant potential. For instance, OGA agonists could work synergistically with OGT or GFAT inhibitors to reduce O‐GlcNAcylation levels, potentially benefiting cancers and CVDs. Conversely, OGT or GFAT agonists could be combined with OGA inhibitors to increase O‐GlcNAcylation levels, offering new approaches for neurodegenerative diseases and some autoimmune disorders. Additionally, antagonistic antibody drugs targeting OGT, OGA, or GFAT are highly promising, as they can block their targets with high specificity and sensitivity, complementing existing inhibitors. Novel drugs regulating the expression of these genes (e.g., siRNA, antisense oligonucleotides) are also worth exploring, particularly for O‐GlcNAcylation‐related diseases caused by abnormal expression.

In addressing the challenge of insufficient spatial specificity, targeted delivery technologies represented by nanomaterials and engineered exosomes offer promising solutions. Various nanomaterials can serve as drug carriers due to their excellent biocompatibility and potential for targeted release. For instance, liposomes—micron‐scale phospholipid vesicles with a bilayer membrane structure—can encapsulate diverse drug molecules and release them in specific organs, making them widely used in interventions for tumors and CVDs [431]. A diverse range of other materials, including nanoparticles, hydrogels, micelles, dendrimers, mesoporous materials, adenoviruses, lysozymes, elastin‐like polypeptides, and chitosan, also demonstrate excellent delivery capabilities [432, 433, 434, 435, 436, 437, 438, 439, 440, 441, 442, 443, 444, 445, 446, 447, 448, 449, 450, 451, 452, 453, 454, 455]. By surface‐modifying these materials and loading them with OGT, OGA, or GFAT inhibitors, high‐precision delivery to specific tissues or lesion sites can be achieved. Exosomes, natural vesicles secreted by cells, play a key role in intercellular communication and material transport. Their low immunogenicity, high biocompatibility, and excellent stability make them ideal drug carriers. Notably, exosomes possess a natural “homing” ability to target diseased areas, and diseased cells (such as tumor cells) produce exosomes in large quantities. Through engineering approaches—loading exosomes with specific inhibitors and modifying their surface molecules—their targeted delivery capacity can be further enhanced, enabling more precise drug delivery to lesion sites. This improves therapeutic efficacy while reducing systemic side effects.

In summary, the main challenges of targeting O‐GlcNAcylation therapeutically are the low efficacy and significant side effects of existing inhibitors, coupled with a lack of tissue or cellular specificity. To overcome these hurdles, future research should focus on three core strategies: iterating and improving upon current drugs; developing novel therapeutics; and utilizing advanced targeted delivery systems for precise spatial drug delivery. Together, these approaches form a critical pathway for the field's advancement.

## 5 Summary

O‐GlcNAcylation is an emerging posttranslational modification that primarily targets Ser/Thr residues, extensively modulating protein structure and function. Since its discovery, it has been consistently shown to play an indispensable role in sustaining normal biological activities and overall health—spanning gene expression, signal transduction, metabolism, and the cell cycle. As a fundamental biological process, gene expression is profoundly shaped by O‐GlcNAcylation. This modification contributes to the assembly of transcription initiation complexes, guides mRNA splicing, and fine‐tunes the initiation, elongation, termination stages of protein translation—thereby exerting precise control over the entire flow of genetic information. In signal transduction, O‐GlcNAcylation exerts significant influence over the activation of diverse signaling cascades and the stability of signaling molecules. For instance, by modifying β‐catenin in the Wnt/β‐catenin pathway, it enhances the protein's stability and thereby supports tissue homeostasis and regeneration. Concurrently, O‐GlcNAcylation regulates RTK pathways such as AKT and MAPK, which are central to cell proliferation, differentiation, apoptosis, and energy storage. Moreover, it can activate cytokine‐related pathways such as TNFα and ILs, enabling cells to adapt to external stimuli and underscoring its critical role in health maintenance. As the core process governing energy supply, metabolism is dynamically regulated by O‐GlcNAcylation. Under energy‐deficient conditions such as fasting, this modification promotes the expression of key gluconeogenic genes—including FOXO1, CRTC2, PGC‐1α, and ERRγ. Conversely, in hyperglycemic environments, it boosts insulin secretion by modulating transcription factors such as PDX1 and NeuroD1. These mechanisms illustrate the central role of O‐GlcNAcylation in maintaining energy balance and metabolic homeostasis. The cell cycle, which underlies growth and proliferation, is strongly supported by O‐GlcNAcylation. It facilitates the orderly progression through G1, S, G2, and M phases and ensures the accurate execution of phase‐specific events. In contrast, deficiency or dysregulation of O‐GlcNAcylation markedly impedes the cell cycle progression, further emphasizing its necessity for cellular proliferation. O‐GlcNAcylation participates broadly and deeply in multiple core biological processes essential for organismal homeostasis. Its multifaceted functions across gene expression, signal transduction, metabolism, and the cell cycle collectively affirm its critical contribution to health and physiological stability.

As research advances, a growing body of data indicates that abnormal elevation or reduction in O‐GlcNAcylation levels is present in various diseases and plays a critical role in their onset and progression. Aberrant O‐GlcNAcylation is widely involved in multiple pathological processes, including CVDs, cancers, immune‐related diseases, neurodegenerative diseases, contributing to disease progression through direct or indirect mechanisms. In CVDs and cancers, O‐GlcNAcylation is often abnormally elevated. In the cardiovascular system, excessively high O‐GlcNAcylation can promote HF by activating the mTOR pathway or induce ventricular cardiomyocyte hypertrophy through PKA pathway activation, significantly exacerbating cardiovascular pathology. In cancers, elevated O‐GlcNAcylation facilitates key processes such as de novo nucleic acid synthesis, oncogene expression, transcription factor activation, CSC formation, immune evasion, and metabolic reprogramming of glucose and lipids, thereby driving the proliferation, invasion, metastasis, and drug resistance of malignancies like lung, liver, colorectal, and breast cancers. Abnormally high O‐GlcNAcylation also plays an important role in immune‐related diseases. Elevated modification levels can lead to dysfunction of T and B cells, enhance their tissue infiltration capacity, and trigger sustained inflammatory responses, thereby promoting conditions such as AIH, MS, RA, and organ transplant rejection. In most neurodegenerative diseases, O‐GlcNAcylation levels are abnormally decreased and similarly contribute to disease progression. Low O‐GlcNAcylation can cause abnormal Tau aggregation in AD, α‐synuclein accumulation and dopamine neuron loss in PD, dysfunction of NPCs in HD, and impaired functions of NF, TDP‐43, and NPGPx in ALS. Notably, in HD, abnormal aggregation of HTT under high O‐GlcNAcylation conditions is also a key pathogenic mechanism. These facts demonstrate that dysregulated O‐GlcNAcylation is a key driver of disease pathogenesis.

O‐GlcNAcylation is significantly dysregulated in a range of diseases. Whether abnormally elevated or reduced, this modification drives disease initiation and progression, establishing it as a crucial pathological regulator. Consequently, targeting O‐GlcNAcylation has emerged as a promising therapeutic strategy. Current approaches to modulate aberrant O‐GlcNAcylation primarily fall into two categories: using OGT or GFAT inhibitors to curb abnormal elevations, or employing OGA inhibitors to augment deficient levels. However, existing inhibitors generally suffer from poor selectivity, off‐target effects, and significant side effects, which limit their clinical application. Additionally, current strategies lack spatial specificity, leading to unintended drug effects in nontarget tissues and compromising both efficacy and safety. To enhance the feasibility and effectiveness of targeting O‐GlcNAcylation, future research should focus on three key strategies: optimizing existing drugs, developing new inhibitors, and utilizing targeted delivery systems. This multipronged approach aims to improve drug performance and local concentration while reducing systemic exposure and related side effects. By integrating drug optimization with innovative delivery systems, O‐GlcNAcylation holds promise as a highly efficient and precise therapeutic target, ultimately accelerating its clinical translation.

Finally, this paper systematically summarizes the beneficial roles of O‐GlcNAcylation in various biological processes and thoroughly analyzes how its dysregulation contributes to disease pathogenesis, thereby elucidating its critical functions in both health and disease. Building on this foundation, we evaluate the feasibility, challenges, and future research directions of targeting O‐GlcNAcylation therapeutically, with the aim of accelerating its clinical translation.

## Author Contributions

Z.R., C.C. and C.Y.: Writing—original draft and visualization. X.L. and C.Y.: Conceptualization, supervision, and review. J.O. and G.W.: Literature collection. All authors have read and agreed to the published version of the manuscript.

## Conflicts of Interest

The authors declare no conflicts of interests.

## Ethics Statement

The authors have nothing to report.

## Funding

This work was supported by the Basic Research Program of Guangzhou Municipal Science and Technology Bureau (Grant number: SL2022A03J00629); the Natural Science Foundation of China (NSFC) (Grant number: 82000714); the Guangdong Medical Science and Technology Research Fund (Grant number: A2024367); and the Plan on Enhancing Scientific Research in GMU (Grant number: GMUCR2024‐01008).

## Data Availability

The authors have nothing to report.
